# Targeted metabolomics of pellicle and saliva in children with different caries activity

**DOI:** 10.1038/s41598-020-57531-8

**Published:** 2020-01-20

**Authors:** Annika Schulz, Roman Lang, Jürgen Behr, Susann Hertel, Marco Reich, Klaus Kümmerer, Matthias Hannig, Christian Hannig, Thomas Hofmann

**Affiliations:** 1 Chair of Food Chemistry and Molecular Sensory Science, Technical University of Munich, Lise-Meitner-Straße 34, D-85354 Freising, Germany; 2 Bavarian Center for Biomolecular Mass Spectrometry, Gregor-Mendel-Straße 4, D-85354 Freising, Germany; 3 Leibniz-Institute for Food Systems Biology at the Technical University of Munich, Lise-Meitner-Straße 34, D-85354 Freising, Germany; 4 Policlinic of Operative and Pediatric Dentistry, Medical Faculty Carl Gustav Carus, Technical University of Dresden, Fetscherstraße 74, D-01307 Dresden, Germany; 5 Sustainable Chemistry and Material Resources, Institute of Sustainable and Environmental Chemistry, Leuphana University of Lüneburg, Universitätsallee 1 C13, D-21335 Lüneburg, Germany; 6 Clinic of Operative Dentistry and Periodontology, Saarland University, Universitätsklinikum des Saarlandes, D-66421 Homburg/Saar, Kirrberger Straße Germany

**Keywords:** Dental caries, Quality of life, Paediatric dentistry

## Abstract

Pellicle is the initial proteinaceous layer that is formed almost instantaneously on all solid surfaces in the oral cavity. It is of essential relevance for any interactions and metabolism on the tooth surface. Up to now, there is no information on the metabolome of this structure. Accordingly, the present study aims to characterise the metabolomic profile of *in-situ* pellicle in children with different caries activity for the first time in comparison to saliva. Small molecules such as carbohydrates, amino acids, organic acids, and fatty acids, putatively involved in the formation of caries were quantified using mass spectrometry (MS)-based techniques, such as (stable isotope dilution analysis)-ultra-performance liquid chromatography-tandem MS and gas chromatography/electron ionisation-MS. Pellicle and corresponding saliva samples were collected from caries-active, caries-free and caries-rehabilitated 4- to 6-year-old children. The most abundant analytes in pellicle were acetic acid (1.2–10.5 nmol/cm^2^), propionic acid (0.1–8.5 nmol/cm^2^), glycine (0.7–3.5 nmol/cm^2^), serine (0.08–2.3 nmol/cm^2^), galactose (galactose + mannose; 0.035–0.078 nmol/cm^2^), lactose (0.002–0.086 nmol/cm^2^), glucose (0.018–0.953 nmol/cm^2^), palmitic acid (0.26–2.03 nmol/cm^2^), and stearic acid (0.34–1.81 nmol/cm^2^). Significant differences depending on caries activity were detected neither in saliva nor in the corresponding pellicle samples.

## Introduction

Despite a general worldwide decline, tooth decay still remains a severe problem for both health and the economy. In 2010, the cost for medical treatment of dental diseases was USD 298 billion worldwide, corresponding to 4.6% of the total global health-care expenditure^1^. Although considered preventable, caries is one of the most prevalent non-communicable diseases in Europe, with up to 100% of the adults having an experience with it^2^. The disease can arise already in the first years of life and challenges the physiological and psychological health of both children and their parents^3,4^. Furthermore, 621 million children are affected worldwide^5^. Up to 26% of 3-year-olds in Germany already show the initial stage of early childhood caries (ECC) or nursing bottle syndrome^6^. ECC is defined as the presence of one or more decayed, missing, or filled tooth surfaces in any primary tooth in a child ages ≤6 years and can be characterised, for example, by the dmf/s = decayed, missing, filled surface (dmf/s) or decayed, missing, filled teeth (dmf/t) index^7^. The development of caries in general and ECC in particular is a multifactorial process^8^, with contributions found in irresponsible feeding practice with unrestricted access to sweetened beverages especially at night, presence of cariogenic microorganisms, genetic factors and in particular inadequate oral hygiene habits^3^. The physical damage of the teeth, e.g. lesions and cavities, is considered the final and visible stage within the progression of this complex illness^9^.

Aside from local pain, untreated caries can successively evolve from lesions further into severe general health issues, affecting children’s body weight gain, growth and overall quality of life^4,10^. Treatment of 3- to 6-year-olds with severe tooth defects often necessitates the complete extraction of the affected teeth under general anaesthesia, which puts a massive strain on young patients. The consequences of such drastic measures may include aesthetic problems, hampered chewing abilities and subsequent malnutrition, and hindered development of speech and articulation due to missing teeth. This can further lead to psychological stress through segregation and stigmatisation by other peers in kindergarten and school, resulting in a substantial loss of quality of life of both children and their parents^11^.

Aside from regular visits to the dentist, the prevention of such scenarios includes educational programmes on oral hygiene at home and diet, on the one hand, and detailed knowledge of factors contributing to the development of caries on a molecular level, possibly even marker compounds indicating initial processes, on the other hand. As saliva is in direct contact with teeth, oral microbiota and bacterial plaque, the analysis of this biofluid may give hints for the detection of caries initiation and progression. Saliva holds valuable analytical information in the context of, for example, drug abuse^12^, diagnosis of pancreatic and oral cancers, prediction of autoimmune diseases, systemic microbial infections and diabetes^13^. For the treatment of very young children, the collection of saliva for diagnostic purposes is reasonable, as it is easily obtained in a non-invasive way without the discomfort of drawing blood^14^. However, because of salivary clearance^15,16^, the metabolome of saliva is transient, with the time of the day, outer temperature and liquid intake substantially affecting its production rate and consistency.

Compared to saliva, pellicle may present a less transient picture of the chemical situation in the mouth, as it adheres to the teeth and is not washed away by saliva. Pellicle is the initial proteinaceous layer formed instantaneously on dental hard and soft tissues by the adsorption of salivary proteins, glycoproteins, and other macromolecules, and it sticks to the tooth surface by forming a layer as a link between the oral fluidic phase and the dental surface^14,17^. Pellicle contains antibacterial components such as lysozyme and peroxidase and protects the teeth to some extent from erosion by lubrication. However, pellicle features also certain binding sites that enable bacterial attachment, which is the basis for dental plaque formation^17,18^. During biofilm formation, different cariogenic and caries-associated microorganisms adhere to pellicle. In particular, *Streptococcus mutans* plays an important role, as this microorganism releases glucosyltransferases into the surroundings and builds insoluble biofilms of glucans. In these physically protected spaces, *S. mutans* and other microorganisms (e.g. *Candida* spp. and *Lactobacilli*)^14^ can flourish in direct contact with the tooth surface and metabolise low molecular carbohydrates into acidic compounds with subsequent tooth demineralisation and formation of initial lesions^9,19,20^. Up to now, reliable, detailed information on the metabolome of pellicle is lacking.

The aim of the present study was to investigate the small metabolites putatively involved, directly or as bacterial substrate, in the formation of caries in children ages ≤6 years to contribute to a better understanding of the physiological and pathophysiological processes in the oral cavity. Carbohydrates, amino acids, organic acids and fatty acids were the targets of quantitative analysis with mass spectrometry (MS)-based techniques, such as [stable isotope dilution analysis (SIDA)]-ultra-performance liquid chromatography (UPLC)-tandem MS (MS/MS) and gas chromatography/electron ionisation (GC/EI)-MS. Saliva and pellicle samples were collected from caries-active, caries-free and caries-rehabilitated children. The metabolome of *in-situ* pellicle was evaluated systematically and quantitatively for the first time.

## Results

The present study aimed to capture the oral metabolomic picture and generate a quantitative data set of the primary metabolites in saliva and oral biofilm samples in children ages 4 to 6 years with different dental states in terms of caries activity. The study participants (n = 57 children) were divided into three groups after a thorough investigation by a dentist and classified as “caries-free” (dmf/t = 0), “rehabilitated” with no active carious lesion (dmf/t ≥ 2; all lesions treated), and “caries-active” (dmf/t ≥ 2; open lesions that need treatment). Table 1 shows the characteristics of the study groups. Children with dental restorations but apparently without active caries were included in the study because this provided an opportunity to evaluate the possible effects of a former active caries infection on subjects with or without active caries.

**Table 1:** Information on study participants, collected data sets of amino acids, organic acids, fatty acids and carbohydrates in saliva and *in-situ* pellicle, and completeness of data (cf. ref. ^22^).

Dental status	Caries free	Rehabilitated	Caries active
Participants, total (male/female)	18 (6/12)	18 (7/11)	21 (11/10)
Age (mean ± SD)	5.5 ± 0.6	5.8 ± 0.7	5.5 ± 0.6
dmf/t	0	9.3 ± 4.4	7.1 ± 2.9
dmf/s	0	30.1 ± 18.7	16.8 ± 11.3
QHI	11.9 ± 7.4	22.1 ± 8.6	35.5 ± 19.5
GI	3.4 ± 3.0	5.8 ± 3.5	7.0 ± 4.8
Saliva samples collected	18 (100%)	18 (100%)	21 (100%)
Amino acids analysed (31/31)^a^	18	18	21
Organic acids analysed (12/22)^a^	18	18	21
Carbohydrates analysed (9/15)^a^	18	16	20
Fatty acids analysed (9/11)^a^	15^b^	12^b^	11^b^
Pellicle samples collected	17 (94%)^c^	13 (72%)^c^	12 (57%)^c^
Amino acids analysed (10/31)^a^	17	12^b^	11
Organic acids analysed (4/22)^a^	17	12^b^	11
Carbohydrates analysed (3/15)^a^	17	12^b^	11
Fatty acids analysed (4/11)^a^	15	13	11

^a^Analytes detected in ≥80% of all samples vs. analytes in the method.

^b^Incomplete data sets due to limited sample volume.

^c^Incomplete data set due to dropouts/no cooperation.

### Sample generation

The parents of the study participants were asked to clean their children’s teeth about 30 min before visiting the dental clinic by tooth brushing and the use of dental floss. Toothpaste, however, was not permitted to elimate toothpaste constituents as possible confounding factors. At the dental clinic the *in-situ-*trials were performed between 9:30–10:00 a.m. During this time, children were not allowed to eat or drink. Unstimulated whole saliva was collected under medical supervision by spitting into sterile tubes, as most of the relevant metabolites show higher abundance in resting saliva compared to stimulated saliva^21^. Samples from all study participants were obtained (100%).

In contrast to this opportune procedure, the collection of pellicle samples initially involved the oral exposition of individually manufactured upper jaw braces containing enamel slabs^22^. However, this method had limited success, as it necessitated at least one additional visit to adjust the braces, and some children felt uncomfortable wearing them during the sampling period, which was needed for biofilm generation. Eventually, pellicle was successfully sampled using custom-made “sampling lollipops” by fixing ceramic slabs (total surface of 8 cm²) onto “lollipop”-shaped silicone forms and placing three per participant buccally into the lower jaw for 10 min. The application of the ceramic material as substrate for pellicle formation is a standardised procedure that has successfully been used previously in a proteomic study on the initial pellicle^23^. The adsorption properties of this material are mainly based on electrostatic interactions, being very similar to tooth enamel, because hydrophilic feldspar ceramic is also negatively charged at physiological pH conditions and has comparable contact angles and the same isoelectric point (3.6 ± 0.2)^24^.

Despite the general acceptance of the lollipops and the higher convenience for patients, sampling succeeded only in 94%, 72%, and 57% of caries-free, dentally rehabilitated and caries-active study participants, respectively (cf. Table 1). The lower sampling rate was mainly caused by the insufficient cooperation from the participants or their parents particularly in the caries-active group.

### Sample preparation and measurement

The preparation steps for the saliva samples were limited to a minimum with the removal of mucins and bacterial debris by centrifugation and proteins by solvent-mediated precipitation. Samples of oral biofilms (pellicle) were thoroughly rinsed with water to remove adherent saliva and subsequently desorbed from the orally exposed ceramic surfaces using Triton-X and radioimmunoprecipitation assay (RIPA) solutions^23^. Clear saliva and pellicle desorbates were spiked with the stable isotope-labelled internal standards (IS) for quantitative purposes and directly analysed by UPLC-MS/MS in the case of amino acids, and after derivatisation in the case of organic acids^25,26^ and carbohydrates^27,28^, respectively, to improve ionisation and discrete fragmentation in MS/MS analysis. Supporting Tables 1–3 contain the ion source and ion path parameters obtained by individual tuning of the analytes and IS as well as their derivatised analogues. The calibration curves for each compound were established with multiple standard solutions in aqueous solvent with the IS given at a fixed concentration and the analytes covering a reasonable concentration range (cf. Supporting Table 4). The chromatographic conditions on reversed-phase-based stationary phases with acidified water and acetonitrile and a hydrophilic interaction liquid chromatography-based phase with NH_4_Ac-buffered and acidified water and acetonitrile enabled sufficient separation to allow the collection of precise and accurate quantitative data. In-house validation results obtained by quantification of the analytes spiked into artificial saliva^29^ are summarised in Supporting Table 4 and comprise information on recovery, precision, linearity of calibration curves an calibrated range, limit of detection and limit of quantification. Representative LC-MS/MS chromatograms of the herein reported methods for the quantification of amino acids, organic acids and carbohydrates in saliva, pellicle and standard solution are provided in the Supporting Information (Supporting Figs. 3–5). Fatty acids (C12–C24) were analysed as fatty acid methyl esters employing a validated and published method using GC/EI-MS^30^.

### Targeted analysis in saliva: data analysis

**Figure 1:**
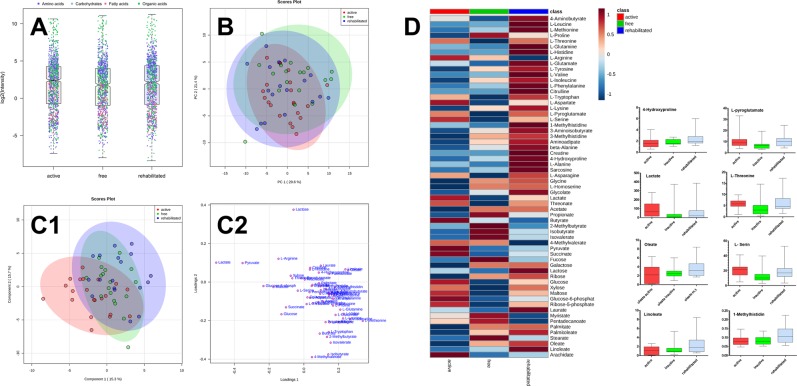
(**A**) Abundance of organic acids (green), amino acids (blue), carbohydrates (grey), and fatty acids (red) in saliva is comparable in the study groups. (**B**) A clear separation of the study groups in the PCA based on targeted data is not possible. (**C**1) A clear separation of the study groups in the PLS-DA based on targeted data is not possible. (**C**2) Loadings plot of PLS-DA. (**D**) Colour-coded values (raw data were “glog-ed” for normalisation) of the group mean concentrations and box-whisker plots (whiskers show min-max and boxes show 25%, 75%, and median) of saliva concentrations (nmol/mL) of the eight top-ranking compounds (ranked based on FDR-adjusted ANOVA; no significant difference between groups was detected). Data were based on 21 carries-active, 18 caries-free, and 18 caries-rehabilitated/medically treated children.

Targeted quantitative data collection of amino acids, organic acids, and carbohydrates by UPLC-MS/MS methods and fatty acids by GC-EI-MS succeeded in both saliva and pellicle samples (cf. Table 1). The qualitative profile was noticeably different between sample types, and only a limited number of analytes was above the quantitation threshold (S/N > 10). In ≥80% of the saliva samples, 31 of 31 amino acids, 12 of 22 organic acids, 9 of 15 carbohydrate derivatives, and 9 of 11 fatty acids were detected. The collected saliva sample sets were complete for all three study groups (100%). In caries-free participants, data sets were complete for amino acids, organic acids, and carbohydrates (all samples were analysed). For caries-active and caries-rehabilitated groups, all samples (100%) were analysed for amino acids, 20 of 21 samples were analysed for organic acids, and 16 of 18 samples were analysed for carbohydrates. Data sets on fatty acids, in general, were incomplete, because only a subset was analysed due to sample limitation (15 of 18 in caries-free participants, 12 of 18 in caries-rehabilitated participants, and 11 of 21 in caries-active participants). Despite substantial inter-individual variance, the quantitatively dominating analytes in all study groups were acetate, propionate and lactate, which ranged from 357 to 2439, 33 to 1295 and 1.5 to 386 nmol/mL, respectively. Amino acids glycine (49–288 nmol/mL) and proline (13–197 nmol/mL) were the most abundant amino acids. Among the carbohydrates, fucose, galactose + mannose (quantified as a sum due to separation issues), and glucose were the most abundant, with 10 to 194, 7 to 194 and 0.8 to 298 nmol/mL, respectively. Oligosaccharides maltotriose, maltotetraose and maltopentaose appeared negligible, as they were detected only in some (<80%) of the samples. Long-chain fatty acids were detected mainly in the lower concentration range. The most abundant analytes in this group were palmitic acid (1–17 nmol/mL), linoleic acid (0.06–8.4 nmol/mL), and oleic acid (0.2–8.4 nmol/mL). Logarithmised data are summarized in Fig. 1A and visualise the enormous spread of individual data and the quantitative dominance of organic acids followed by amino acids and carbohydrates.

The data sets of saliva samples were analysed by one-way analysis of variance (ANOVA) followed by post-hoc analysis (Fisher’s least significant difference) for comparison of the study groups. Missing values (6.8%) due to incomplete fatty acid data were imputed by the k-nearest neighbour (KNN) algorithm (available at http://www.metaboanalyst.ca). However, this resulted in neither a clear separation nor significant differences between the groups, as it became evident from the nearly congruent colour areas [95% confidence intervals (95% CIs)] in the principal component analysis (PCA) scores plot in Fig. 1B. In fact, the PCA scores plot suggested that the caries-rehabilitated samples were scattered within the other two groups, showing properties of both caries-active and caries-inactive groups. Partial least squares-discriminant analysis (PLS-DA; Fig. 1C1) aimed at maximising covariance did not allow a clear separation of the groups, but some hints that compounds contributing to the small differences could be taken from the loadings plot (Fig. 1C2; e.g. lactate and pyruvate). Although no significantly different compounds were obtained by ANOVA [false discovery rate (FDR)-adjusted q-values were ≥0.168], a ranking of the most important analytes included lactate, L-serine, L-pyroglutamate, 4-hydroxyproline, 1-methylhistidine and L-threonine (cf. the ranking in Supporting Table 5) as important analytes. Figure 1D shows the colour-coded group means of the analytes. Figure 1D further shows the quantitative data of the top eight compounds, which were quantitatively different but did not reach statistical significance after FDR adjustment, as box-whisker plots.

We repeated the analysis for the separation of the study groups, including (A) all participants but only amino acids, organic acids, and carbohydrates (missing values 2% were KNN imputed) and (B) all analytes but only those study participants with complete data sets (all compound classes were analysed; missing values 2.2% were KNN imputed; cf. Supporting Figs. 1 and 2). However, although no improved separation of the study groups and no significant markers with p < 0.05 were obtained, the top-ranking (lowest FDR-adjusted p value) analytes were (A) L-serine, L-pyroglutamate, 4-hydroxyproline, glucose, L-threonine, and 1-methylhistidine (FDR ≥ 0.143) and (B) pyruvate, glucose-6-phosphate, L-serine, L-threonine, glucose, L-pyroglutamate, and maltose (FDR ≥ 0.138; cf. Supporting Table 6).

### Targeted analysis in pellicle: data analysis

**Figure 2:**
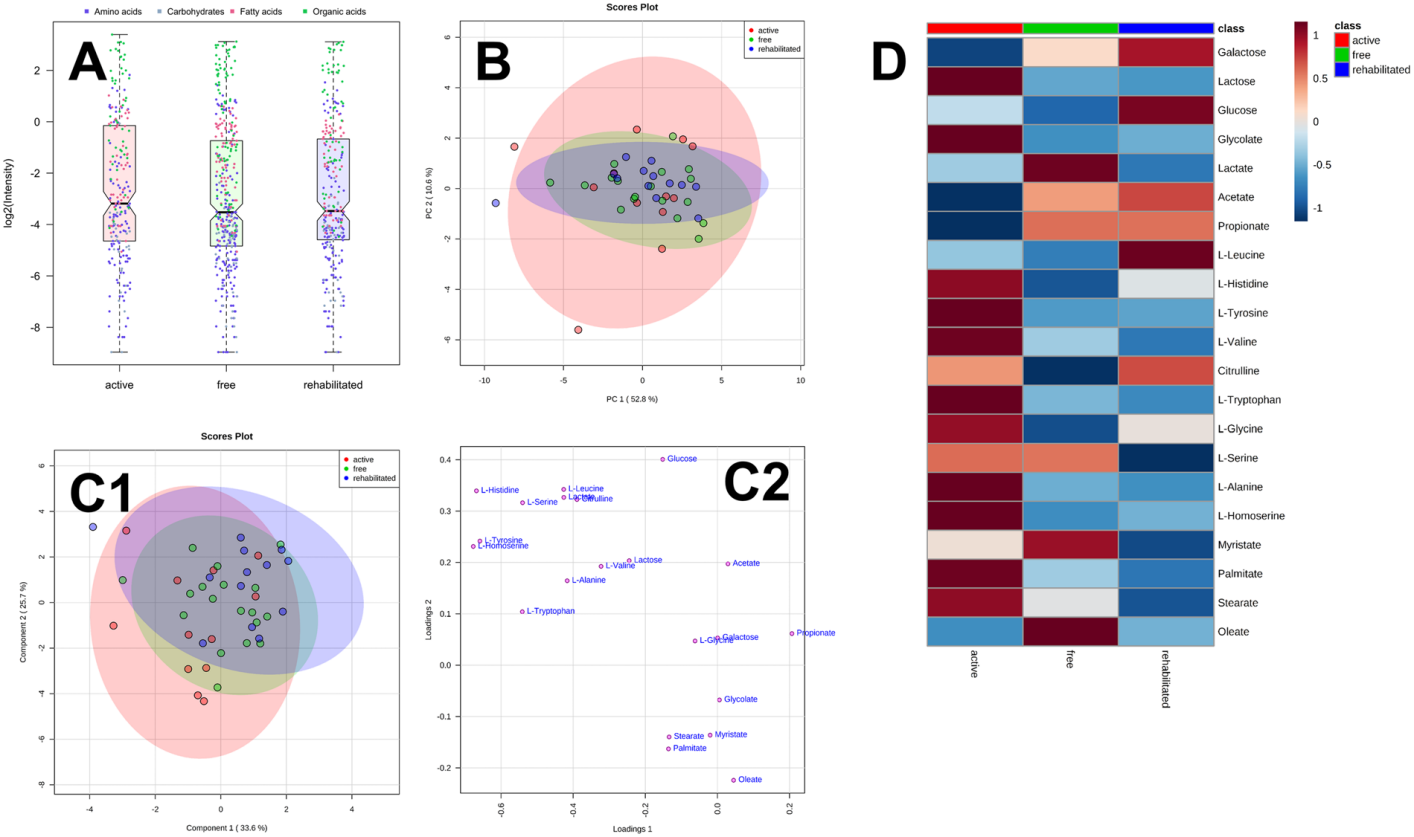
(**A**) Abundance of organic acids (green), amino acids (blue), carbohydrates (grey), and fatty acids (red) in pellicle is comparable in the study groups. (**B**) A clear separation of the study groups in the PCA based on targeted data is not possible. (**C1**) A clear separation of the study groups in the PLS-DA based on targeted data is not possible. (**C2**) Loadings plot of PLS-DA. (**D**) Colour-coded values (raw data were “glog-ed” for normalisation) of the group mean concentrations. Data were based on 12 carries-active, 18 caries = free, and 13 caries-rehabilitated/medically treated children.

Targeted analysis in desorbed samples of oral biofilm succeeded for 11 of 31 amino acids, 4 of 22 organic acids, 3 of 15 carbohydrates and 4 of 11 fatty acids. The pellicle data sets were nearly complete for all compound classes. However, only 13 of 18 caries-rehabilitated and 12 of 21 caries-active samples were available due to dropouts and/or lacking cooperation from the parents. In the pellicle matrix, the qualitative and quantitative abundance of target molecules was by far lower compared to saliva. However, dominating analytes again were found in the group of acidic compounds, namely acetate (1.2–10.5 nmol/cm^2^) and propionate (0.1–8.5 nmol/cm^2^), followed by amino acids glycine and serine, ranging from 0.7 to 3.5 and from 0.08 to 2.3 nmol/cm^2^, respectively. In contrast to saliva, proline was detected in only 8 pellicle samples, in which the highest concentration was 0.1 nmol/cm^2^. Only traces of carbohydrates were detected, in which just galactose (galactose + mannose; 0.035–0.078 nmol/cm^2^), lactose (0.002–0.086 nmol/cm^2^), and glucose (0.018–0.953 nmol/cm^2^) were quantifiable in ≥80% of the samples. Palmitic acid and stearic acid were the most abundant long-chain fatty acids ranging from 0.26 to 2.03 and from 0.34 to 1.81 nmol/cm^2^, respectively. The logarithmised quantitative data are summarized in Fig. 2A. Similar to saliva, the quantities of analytes in pellicle samples showed a large spread, with organic acids showing highest abundance followed by amino acids and carbohydrates. PCA and PLS-DA of the data acquired by targeted analysis gave strongly overlapping 95% CIs (Fig. 2B and C1) and no identifiable compound pattern that could be attributed to one of the three conditions (caries active, caries inactive and caries rehabilitated). Figure 2D shows the group means of the data (detected in ≥80% of individuals per group). ANOVA with FDR adjustment indicated no significant differences between groups in individual analytes (cf. Supporting Table 7 for the FDR-adjusted ranking of the compounds).

## Discussion

The aim of the study was to gain quantitative information on the abundance of small metabolites, organic acids, amino acids, carbohydrates and fatty acids in samples of saliva and initial pellicle obtained from caries-active, caries-inactive and caries-rehabilitated children ages ≤6 years and possibly link the quantitative data to the oral health condition.

The targeted data obtained from saliva samples by dedicated UPLC-MS/MS methods were investigated by ANOVA. The analytes in saliva with the most considerable influence on the separation of the study groups identified by the smallest FDR-corrected p-values were lactate, L-serine, L-pyroglutamate, 4-hydroxyproline, 1-methylhistidine, L-threonine, oleate and linoleate, albeit no significant differences were detected.

Lactate and 1-methylhistine are two of the metabolites that emerged in the literature previously in the context of caries activity^31,32^. However, data are conflicting, as increased lactate concentrations in saliva of caries-active children have been reported^32^ but not confirmed^33^. *In vitro* experiments revealed that lactate exposition leads to tooth enamel demineralisation to a greater extent than acetate or propionate^34^. However, given the fact that acidic conditions are necessary, the strong buffering capacity of saliva^35^ may literally neutralise this issue *in vivo*. In this study, lactate concentrations in caries-active children appeared elevated compared to the other two groups, but the inter-individual spread of the data was very large and strongly overlapping. Mean ± standard deviation (SD) values were 88 ± 84, 40 ± 89, and 65 ± 104 mM for caries-active, caries-inactive and caries-rehabilitated children, respectively. The intake of food and drinks within the study day might substantially contribute to organic acids in saliva, although during sampling sessions neither eating nor drinking was allowed^36^. This applies particularly to lactate, which originates from bacterial carbohydrate metabolism following the Embden-Meyerhof-Parnas pathway^37,38^ and thus may confound the findings. Although the study participants (children ages ≤6 years) were asked to abstain from food or drink intake before sample collection, we were unable to check their compliance. Furthermore, given the good solubility of lactate in aqueous systems, we expect saliva clearance to affect the abundance of both lactate and other soluble compounds in the oral cavity.

Further metabolites identified by ANOVA, such as L-serine, L-pyroglutamate, 4-hydroxyproline, 1-methylhistidine and L-threonine, all belong to the group of amino acids. L-serine, L-pyroglutamate and L-threonine showed lower concentrations in the inactive group than in the other two groups, whereas it was opposite for the other two compounds. For nine other amino acids (alanine, aspartate, glutamine, glycine, isoleucine, leucine, proline, taurine, and tyrosine), an increased concentration in swap-collected unstimulated saliva of caries-active children has been shown^33^. However, our data did not confirm this. High levels of free amino acids in caries-active children have been linked to increased protein hydrolysis activity by bacteria^39^ and a high abundance of proline and glycine as a possible result of the hydrolysis of particularly dentin-collagen^40^. In contrast, proline, glycine or 4-hydroxyproline concentrations were not increased in caries-active children compared to healthy ones in our study. Elevated concentrations of stearate and reduced oleate concentrations have been reported in caries-susceptible adults’ parotid saliva^41^. In children, a similar picture was obtained in whole saliva samples, as stearate was 4.7 ± 1.6, 2.8 ± 1.5, and 3.7 ± 2.1 µM in caries-active, caries-inactive and caries-rehabilitated children, respectively.

Although neither significant differences between the three groups nor a correlation with their oral health status were detected, it was striking to see that, in the PCA scores plot, caries-rehabilitated children were scattered within the caries-active and caries-inactive groups. In fact, this can be interpreted in different ways. (A) Although the targeted data in this paper provide an analytical picture of amino acids, organic acids, fatty acids and carbohydrates in caries-active, caries-inactive and caries-rehabilitated children, none of the compounds is a major contributor to tooth decay. Therefore, caries-related investigations can proceed with analyses of middle and high molecular weight components such as peptides and proteins and non-targeted metabolomic approaches. (B) The nature of saliva production and clearance does not allow data acquisition of significant caries-related analytes due to the lack of accumulation of important small molecules.

Despite the advantage that biofluid saliva offers convenience of collection, it appears that a single saliva sample obviously does not deliver reliable markers of caries activity due to saliva clearance, as it is just a short snapshot of the current circumstances. Furthermore, the metabolite composition of saliva is influenced by diverse physiological and environmental factors^42^. Using children as test participants, most of these effects, e.g. smoking, are circumvented, but gender or collection time can also affect the metabolite profiles^42^. Dilution effects generated by the normal salivary process of producing and swallowing saliva do not permit an accumulation of metabolites. Punctual enrichments of produced metabolites on the tooth surface, e.g. acids produced by bacterial microorganisms, are apparently immediately washed away by the salivary flow. Moreover, potential differences in saliva composition are diluted by the normal flow of saliva during the day. For future studies, sample collection directly after waking up in the morning to benefit from overnight accumulation of metabolites due to decreased saliva production in the night should be considered.

Aside from analysis of children’s saliva samples, similar analytical approaches were performed for their pellicle samples, which were expected to display a more stable picture. The analysis of pellicle for diagnostic purposes has increased over the last years, as former limitations, such as small sample amounts, appear manageable by the development of efficient pellicle collection methods and increased sensitivity of analytical instruments^23^. The pellicle layer is a valuable analytical target because marker metabolites might be enriched on the tooth surface where the caries infection starts.

As the most abundant metabolites, acetic acid, propionic acid, glycine, serine, galactose + mannose, lactose, glucose, palmitic acid and stearic acid were quantified. These data fit to previous studies about pellicle in adult subjects. Glucose was reported as the most abundant carbohydrate in the pellicle matrix^43^.

Glucans, synthesised by glucosyltransferases, and glycolipids containing glucose as the carbohydrate moiety have been discussed as possible sources for glucose in pellicle^17^. Interestingly, the investigation of the amino acid composition after hydrolyses with hydrochloric acid led to the same results as in the present study. Previous studies stated serine and glycine as the most abundant amino acids and significant lower levels of proline in pellicle compared to saliva^44,45^. Furthermore, palmitic acid and stearic acid have previously been reported as the most abundant fatty acids in pellicle of adult test participants^46^.

The comparison of quantitative data showed no significant differences between the metabolite profiles of pellicle of caries-free, caries-rehabilitated and caries-active children. In total, only 22 analytes were detected in ≥80% of the samples per group; therefore, it was not possible to select single analytes that contributed to a categorisation based on caries status. We expected hardly any bacterial colonisation of the initial pellicle formed within a formation time of 10 min, so the data presented here refer to the initial pellicle composition. We thus assume that the detected metabolites in pellicle were either synthesized by endogen enzymes in pellicle or adsorbed into pellicle from the surrounding saliva. However, no evidence was found that linked the investigated metabolites to bacterial activity, as no separation of the three study groups due to their caries status was possible. The general metabolic conditions in pellicle therefore seem to be very similar among all subjects. Based on the data obtained in this study, we think that a longer formation time, which might result in an accumulation of metabolites, can produce more meaningful results. Furthermore, after a longer incubation time in the oral cavity, differences due to bacterial adhesion could possibly be detected, e.g. an accumulation of organic acids produced by microorganisms or altered concentrations of carbohydrates due to the hydrolytic cleavage of glycoproteins by bacterial enzymes^33^. However, from our experience, incubation times of more than 10 min for pellicle collection within a study of children ages ≤6 years is hard to realise.

A recent study on the activity and distribution of pellicle enzymes in children did not find significant differences in enzyme activity related to the children’s caries activity^22^. This finding supports the present results, which seems unlikely to identify metabolites in short-term pellicle as markers for caries activity. Although pellicle, in contrast to saliva, appears to provide a more stable picture of the processes in the oral cavity, some difficulties within the search for metabolites as marker substances in this matrix need to be considered. Rinsing of the ceramic surfaces after biofilm formation is necessary to remove adherent saliva residues. However, the fact that water-soluble metabolites might be washed off, thus considerably affecting concentrations, poses a serious problem for quantitative analysis. Furthermore, surfactant-mediated desorption of the biofilm from ceramic specimens results in a dilution step, which makes the analysis of the already very low concentrated metabolites even more challenging.

The distribution of the samples in the PCA scores plot showed that caries-active samples are widely spread and therefore not showing a homogenous picture. This scattering of caries-active subjects suggested that there is not one typical metabolite profile of the 10-min pellicle that represents caries or that, as already discussed, the analysed metabolites in pellicle are not suitable to represent the reliance on caries.

Comparing saliva and pellicle data, 17 of 70 analysed substances were detected in ≥80% of all saliva and pellicle samples and therefore used for Spearman correlation testing. About 76% of the correlation coefficients were smaller than 0.2, indicating very weak correlation between these metabolites in saliva and pellicle. The remaining analytes showed correlation coefficients between 0.2 and 0.5, which was classified as a weak correlation. Therefore, the analysis of saliva cannot be used as a prediction of metabolite concentrations in pellicle and vice versa. The formation of pellicles seems to be a very specific process. Not all analytes found in saliva are detectable in pellicle, and the ratio of analytes also differs in both matrices. As already mentioned, proline is an interesting example. This amino acid was quantified in all saliva samples and had the second highest concentration (12.5–197.4 nmol/mL) in total, whereas this analyte was quantified only in 20% of pellicle samples and just in minor concentrations (≤1 nmol/cm^2^). These findings support the thesis that metabolites are not merely adsorbed into pellicle according to their concentration in saliva or are not metabolised in pellicle to the same extent as in saliva. A study on proteins and peptides in saliva and pellicle also showed differences in the composition of both matrices for these substance classes, possibly indicating adsorption of proteins/peptides from oral sources other than saliva and representing pellicle formation as a process that is not entirely dependent on salivary components^47^. Also, the fatty acid profile of pellicle differs from that of saliva. This indicates that fatty acids in saliva are not simply adsorbed on the tooth surface according to their availability or concentration^46^. Interestingly, the higher amount of bacterial biofilm and the more pronounced gingivitis in caries-active and caries-rehabilitated children, respectively [cf. Quigley Hein index (QHI) and gingival index (GI) in Table 1], did not result in quantitative or qualitative differences in the metabolome of saliva and pellicle.

Summarising the data, for the first time, the pellicle metabolome in children, as well as the corresponding saliva samples, was systematically investigated and comprehensively characterised with targeted (SIDA)-UPLC-MS/MS and GC/EI-MS. The most abundant analytes in pellicle formed within 10 min were acetic acid (1.2–10.5 nmol/cm^2^), propionic acid (0.1–8.5 nmol/cm^2^), glycine (0.7–3.5 nmol/cm^2^), serine (0.08–2.3 nmol/cm^2^), galactose (galactose + mannose; 0.035–0.078 nmol/cm^2^), lactose (0.002–0.086 nmol/cm^2^), glucose (0.018–0.953 nmol/cm^2^), palmitic acid (0.26–2.03 nmol/cm^2^), and stearic acid (0.34–1.81 nmol/cm^2^). Significant differences due to caries activity were obtained in neither saliva nor pellicle metabolome regarding the most discussed metabolites amino acids, organic acids, fatty acids and carbohydrates. Thus, pellicle as an important biological mediator in the oral cavity seems to be independent of caries status. These results reinforce the observations of pellicle formation as a unique and either very selective or chaotic process, with quite complex and inter-individual variance even after very short formation time.

## Materials and Methods

### Chemicals

Solvents were of LC-MS grade (Honeywell Riedel-de Haën, Seelze, Deutschland). Water was prepared by a Milli-Q apparatus (Millipore, Schwalbach, Germany). Chemicals were purchased from Sigma-Aldrich (Steinheim, Germany) and Merck (Darmstadt, Germany). Stable isotope-labelled compounds were purchased from Cambridge Isotope Laboratories (Radeberg, Germany) and Sigma-Aldrich. Artificial saliva for method development and validation was prepared according to the literature^29^. In addition, artificial saliva was prepared without α-amylase for method development of carbohydrates, because α-amylase gave a blank value for glucose and maltose of substantial peak area.

### Subjects

A total of 57 children (33 female and 24 male volunteers ages 4–6 years) participated in the present study. Saliva for the analysis of amino acids, carbohydrates, and organic acids was collected from all subjects, in which 18 were caries free with no decayed, missing, or filled teeth (dmf/t = 0), 18 had dental restorations (dmf/t ≥ 2; no active carious lesions), and 21 were caries active (dmf/t ≥ 2; at least two carious lesions). Pellicle samples were collected from 40 children out of the panel, in which 17 were caries free (dmf/t = 0), 12 had dental restorations (dmf/t ≥ 2), and 11 were caries active (dmf/t ≥ 2). The analysis of long-chain fatty acids was performed in saliva and pellicle samples of 40 children, in which 16 were caries free (dmf/t = 0), 13 had dental restorations (dmf/t ≥ 2), and 11 were caries active (dmf/t ≥ 2). Oral examination was conducted by a dentist experienced in paediatric dentistry. The following indices were determined: dmf/t, dmf/s, QHI and GI.

See Table 1 for details on the study groups. The study design was approved by the Ethics Committee of the Technische Universität Dresden (EK #79032012, Ethics Committee of the Medical Faculty, TU Dresden) before recruitment and study initiation. The study was performed according to the guidelines of the Declaration of Helsinki. The clinical part and the acquisition procedures of saliva and pellicle samples, respectively, were checked and approved by the local ethics committee. Informed written consent was obtained from all participants and their legal guardians.

### Saliva and pellicle collection

The parents were asked to clean their children’s’ teeth in the morning and 30 min before pellicle collection by tooth brushing without toothpaste, and application of dental floss. Around 10:00 am unstimulated saliva was collected under medical supervision by spitting in a sterile tube. The samples were immediately frozen −20 °C and then stored at −80 °C until further sample preparation.

For pellicle collection, ceramic specimens were fixed to “lollipops” made of two-component silicone (nine specimens per lollipop). The lollipops were placed in the buccal cavity in the lower jaw, in which the specimens were faced towards the tongue. In this way, three lollipops were incubated in the oral cavity one after the other for 10 min each for pellicle formation. The acquired pellicle was desorbed by working at a clean bench with sterile materials using an in-house-developed protocol. Triton-X buffer consisting of 10% 10 × Tris-HCl-NaCl buffer (0.2 M Tris, 1.5 M NaCl, pH 7.5), 20% protease inhibitor (cOmplete, EDTA free; Roche, Mannheim, Germany), and 1% Triton-X in bidistilled H_2_O and RIPA buffer consisting of 10% 10 × RIPA buffer (Cell Signaling Technologies, Frankfurt, Germany) and 20% protease inhibitor (cOmplete, EDTA free, Roche, Mannheim, Germany) in bidistilled H_2_O were prepared for desorption. The specimens were rinsed with ultrapure water (20 mL) to remove loosely attached salivary components. Pellicle was subsequently desorbed by rinsing with Triton-X buffer (700 µL) and RIPA buffer (700 µL). Both solutions were pooled and stored at −80 °C until further sample preparation. Pellicle collection for the analysis of long-chain fatty acids was done according to the literature with a pellicle formation time of 30 min followed by desorption with 0.4% EDTA solution^30,46^.

The collection of pellicle and saliva samples was done during a single session. During the session, participants refrained from eating and drinking.

### Quantitative analysis

Calibration standards for amino acids, organic acids, and carbohydrates were prepared individually per compound class from diluted mixtures of stock solutions of the unlabelled substances and mixed with a fixed amount of labelled compound as the IS according to the calibration ranges and protocols detailed below. Calibration standards were injected as triplicates. The area ratios of the analyte/IS were plotted against the concentration ratios followed by linear regression to create the calibration curves. For in-house validation, artificial saliva was spiked with a mix of the analytes in final concentrations of 1, 10, and 100 nmol/mL. The samples were processed in triplicate according to the respective quantitation protocol (see below). Each sample was analysed in replicates (n = 3), and to evaluate the technical precision, one sample was injected 10 times. The ratio of calculated to nominal concentration × 100% represents accuracy, and precision was expressed as relative SD (cf. Supporting Table 4).

The analysis of fatty acids in saliva and pellicle desorbates was performed as described in detail previously^30,46^.

### Amino acids

#### Stock solutions of analytes and IS and calibration

Unlabelled amino acids were individually dissolved in 30% aqueous acetonitrile. Appropriate aliquots were combined to obtain a mixed analyte solution (1000 nmol/mL). Labelled amino acids were combined in a mixed IS solution with concentrations between 50 and 250 nmol/mL (see details in Supporting Table 8). Dilutions of the analyte solution with 90% acetonitrile were spiked with the IS solution (100 µL) to obtain final analyte concentrations of 100, 50, 25, 10, 5, 2.5, 1, 0.5, 0.25, 0.1, 0.05, 0.025, 0.01, 0.005, 0.0025, and 0.001 nmol/mL of each analyte with a fixed amount of IS.

#### Sample preparation for quantitation

Saliva samples and pellicle desorbates were centrifuged (10 min, 4 °C, 12,000 rpm) to separate mucins. The supernatant (100 µL) was mixed thoroughly with the IS (5–25 nmol/mL, 10 µL) in an Eppendorf cap (2 mL). Ice-cooled acetonitrile (390 µL) was added and the solution was mixed again. The precipitate was separated by centrifugation (10 min, 4 °C, 13,200 rpm), and the supernatant was transferred into another Eppendorf cap and evaporated to dryness (37 °C, 90 min, 35 kPa). The residue was solved in 50% aqueous acetonitrile (50 µL) and stored at −20 °C until measurement.

#### Instrumental analysis with UPLC-MS/MS

The amino acids were separated on an Acquity BEH amide column (1.7 µm, 2.1 × 100 mm, Waters, Eschborn, Germany) at a flow rate of 0.4 mL/min. Water containing ammonium acetate (5 mM) and formic acid (0.1%) was solvent A, and 95% acetonitrile with ammonium acetate (5 mM) and formic acid (0.1%) was solvent B. The gradient elution started with 90% solvent B for 0.02 min. Solvent B was decreased to 85% within 4.98 min and 70% within 3 min. Solvent A was increased to 100% within 1 min followed by isocratic elution (2 min). The re-establishment of the starting conditions was done within 1 min followed by re-equilibration time for 2 min. The UPLC System (Shimadzu Nexera X2; Shimadzu, Duisburg, Germany) was coupled to a 6500 Q-Trap MS (Sciex, Darmstadt, Germany) with Analyst 1.6.2. Ionisation was done in positive electrospray mode. The configuration was 35 psi for curtain gas, 5500 V for ion spray voltage, 55 psi for heater gas, and 65 psi for turbo gas. The source temperature was set to 400 °C. MS analysis was done by means of a scheduled MRM method. See Supporting Table 1 for MS/MS parameters.

### Organic acids

#### Reagents, stock solutions of analytes and IS, and calibration curves

Stable isotope-labelled organic acids were mixed in 50% acetonitrile at a final concentration of 2000 nmol/mL. A mixture of unlabelled standards was prepared in 50% acetonitrile at a concentration of 2000 nmol/mL. The standard mixture of organic acids with a concentration of each analyte of 2000 nmol/mL was serially diluted 1:1 with water leading to solution from 1000 to 0.25 nmol/mL per analyte. Aliquots (50 µL) of each standard solution were mixed with the IS working solution (200 nmol/mL, 50 µL). This resulted in 14 standards with a fixed amount of each stable isotope-labelled standard and 1000, 500, 250, 125, 62.5, 31.25, 15.6, 7.8, 3.9, 1.96, 0.98, 0.49, 0.25, and 0.125 nmol/mL of each analyte. Solutions of 3-nitrophenylhydrazine (3-NPH; 200 mM in ACN/H_2_O) and N-(3-dimethylaminopropyl)-N′-ethylcarbodiimide (EDC; 120 mM in ACN/H_2_O containing 6% pyridine) were prepared for derivatisation following the literature protocol^25,26^. Prepared calibration standards were treated similar to authentic saliva samples as described in detail below.

#### Sample preparation for quantitation

Saliva samples and pellicle desorbates were centrifuged (10 min, 4 °C, 12,000 rpm) and the supernatant (100 µL) was mixed with the IS solution (200 nmol/mL, 50 µL). Ice-cooled acetonitrile (350 µL) was added and precipitated proteins were removed by centrifugation (10 min, 4 °C, 13,200 rpm). Solutions of 3-NPH (20 µL) and EDC (20 µL) were added to the supernatant and incubated (30 min, 40 °C) after mixing. Water (460 µL) was added and the solution was centrifuged (3 min, 10 °C, 12,000 rpm). Note: The samples should be measured immediately or stored at −20 °C. Precipitates can occur upon storage, which need to be removed (e.g. centrifugation) before instrumental analysis.

#### Instrumental analysis with UPLC-MS/MS

For the chromatographic separation of organic acids, an Acquity BEH C18 column was used (1.7 µm, 2.1 × 100 mm; Waters, Eschborn, Germany). The flow rate was set to 0.4 mL/min, and 0.1% formic acid in water (solvent A) and acetonitrile (solvent B) served as solvents. The gradient started with an isocratic step at 7% solvent B (2 min) and increased to 45% (7.5 min) and then to 100% solvent B (0.5 min). After isocratic elution (1 min), the starting conditions we re-established (0.1 min) followed by the equilibration of the system at 7% solvent B (2.9 min). The UPLC System (Shimadzu Nexera X2, Shimadzu, Duisburg, Germany) was coupled to a 5500 Q-Trap MS (Sciex, Darmstadt, Germany) with Analyst 1.6.2. Ionisation was performed in negative electrospray mode. The setting was 35 psi for curtain gas, −4500 V for ion spray voltage, 55 psi for heater gas, and 65 psi for turbo gas. The source temperature was set to 500 °C. MS/MS analysis was done in MRM mode, in which each transition was recorded with a dwell time of 5 ms. See Supporting Table 2 for MS/MS parameters.

### Carbohydrates

#### Reagents, stock solutions of analytes and IS, and calibration curves

The IS ^13^C_6_-glucose and ^13^C_5_-ribose were mixed in 50% aqueous acetonitrile to a final concentration of 50 nmol/mL. The unlabelled compounds were mixed in 50% aqueous acetonitrile at a final concentration of 2000 nmol/mL. The analyte solution was appropriately diluted with 10% acetonitrile to 500, 100, 50, 10, 5, 1, 0.5, 0.1, 0.05, 0.01, 0.005, and 0.001 nmol/mL of each analyte. Aliquots (100 µL) were mixed with the IS solution (5 µL). This resulted in 12 standards with a fixed amount of each stable isotope-labelled standard. Each standard solution was derivatised. 2-Anthranilic acid (2-AA; 0.35 M in DMSO containing 15% glacial acetic acid) and 2-picoline borane complex (2-PB; 1 M in DMSO) were prepared for derivatisation^28^.

#### Sample preparation for quantitation

Saliva samples were centrifuged (10 min, 4 °C, 12,000 rpm) and a mixture of stable isotope-labelled standards (50 nmol/mL, 5 µL) was added to the supernatant (100 µL). By adding ice-cooled acetonitrile (395 µL) and centrifugation (10 min, 4 °C, 13,200 rpm), proteins were precipitated and separated. The supernatant was transferred into another Eppendorf cap and evaporated to dryness (37 °C, 70 min, 35 kPa). The residue was solved in 10% aqueous acetonitrile (100 µL) and mixed with solutions of 2-AA (5 µL) and 2-PB (5 µL) for derivatisation. After the reaction (2 h, 65 °C), the mixture was centrifuged (10 min, 10 °C, 13,200 rpm), and the supernatant was transferred into a vial and stored at −20 °C until measurement.

Centrifuged pellicle desorbate (100 µL) was spiked with the IS solution (50 nmol/mL, 5 µL) and mixed with ice-cooled acetonitrile (395 µL). After centrifugation (10 min, 4 °C, 13,200 rpm), the supernatant was evaporated, and the residue was taken up in 10% aqueous acetonitrile (200 µL) and derivatised. After the reaction (2 h, 65 °C), the turbid solution was transferred into centrifugal filter units (Amicon Ultra, 0.5 mL Centrifugal Filters, Ultracel 3 K, Regenerated Cellulose; Merck) and centrifuged (10 min, room temperature, 12,000 rpm). The clarified supernatant was transferred into a vial and stored at −20 °C.

#### Instrumental analysis with UPLC-MS/MS

Separation was achieved with a Kinetex F5 column (1.7 µm, 2.1 × 100 mm; Phenomenex, Aschaffenburg, Germany). The flow rate was set to 0.4 mL/min, and 0.1% formic acid in water (solvent A) and acetonitrile (solvent B) were used as solvents. The gradient started at 5% solvent B and increased to 6% (6 min) and then to 100% solvent B (1 min). After isocratic elution (2 min), the re-establishment of the starting conditions was done (0.5 min) followed by an equilibration time of 3.5 min at 5% solvent B. The UPLC System (Shimadzu Nexera X2, Shimadzu, Duisburg, Germany) was coupled to a 5500 Q-Trap MS (Sciex, Darmstadt, Germany) with Analyst 1.6.2. Ionisation was performed in negative electrospray mode. The setting was 35 psi for curtain gas, −4500 V for ion spray voltage, 55 psi for heater gas, and 65 psi for turbo gas. The source temperature was set to 500 °C. For MS/MS analysis, the MRM mode was used, in which each transition was recorded with a dwell time of 20 ms. See Supporting Table 3 for MS/MS parameters.

#### Statistics

Data handling was done using Microsoft Excel 2016, GraphPad 5.0 for Windows (GraphPad Software, San Diego, CA, USA), and MetaboAnalyst 4.0 (https://www.metaboanalyst.ca/)^48–50^. For statistical evaluation, analytes that could be quantified (S/N > 10) in more than 80% of the samples per group were used. The data were log transformed. To evaluate the significant differences among the caries-free, caries-rehabilitated, and caries-active groups, univariate analysis was performed by one-way ANOVA. The FDR was set to default (0.05). Further information is found in the figure descriptions.

## Supplementary information

Targeted Metabolomics of pellicle and saliva in children with different caries activity.

Targeted Metabolomics of pellicle and saliva in children with different caries activity SI Saliva.

Targeted Metabolomics of pellicle and saliva in children with different caries activity SI Pellicle.

## Data Availability

All data generated or analysed during this study are included in this published article (and its Supplementary Information Files).
