# Supplementary material for: Targeted metabolomics of pellicle and saliva in children with different caries activity
Source: Sci Rep. 2020 Jan 20;10:697. doi: 10.1038/s41598-020-57531-8 (PMC6971297; doi:10.1038/s41598-020-57531-8)
Supplement: Supplementary file 1 — Targeted Metabolomics of pellicle and saliva in children with different caries activity. [file 41598_2020_57531_MOESM1_ESM.docx]

**Targeted Metabolomics of pellicle and saliva in children with different caries activity**

**Supporting Information**

Annika Schulz^1^, Roman Lang^1,*^, Jürgen Behr^2,3^, Susann Hertel^4^, Marco Reich^5^, Klaus Kümmerer^5^, Matthias Hannig^6^, Christian Hannig^4^, Thomas Hofmann^1,2,3,*^

^1^ Chair for Food Chemistry and Molecular Sensory Science, Technische Universität München, Lise-Meitner-Straße 34, D-85354 Freising, Germany

^2^ Bavarian Center for Biomolecular Mass Spectrometry, Gregor-Mendel-Straße 4, D-85354 Freising, Germany

^3^ Leibniz-Institute for Food Systems Biology at the Technical University of Munich, Lise-Meitner-Straße 34, D-85354 Freising, Germany

^4^ Policlinic of Operative and Pediatric Dentistry, Medical Faculty Carl Gustav Carus, Technische Universität Dresden, D-01307 Dresden, Germany

^5^ Sustainable Chemistry and Material Resources, Institute of Sustainable and Environmental Chemistry, Leuphana University of Lüneburg, Universitätsallee 1 C13, DE-21335 Lüneburg, Germany

^6^ Clinic of Operative Dentistry and Periodontology, Saarland University, Universitätsklinikum des Saarlandes, D-66421 Homburg/Saar, Germany

^*^ To whom correspondence should be addressed

## PHONE +49-8161/71-2902 (T.H.)

+49-8161/71-2978 (R.L.)

## FAX +49-8161/71-2949

##

## E-MAIL thomas.hofmann@tum.de

roman.lang@tum.de

**content**

- **Supporting table 1**: Ionsource and path parameters for the detection of amino acids by UPLC-MS/MS in positive Electrospray (scheduled MRM).
- **Supporting table 2**: Ionsource and path parameters for the detection of derivatized organic acids by UPLC-MS/MS in negative Electrospray.
- **Supporting table 3**: Ionsource and path parameters for the detection of derivatized carbohydrates by UPLC-MS/MS in negative Electrospray.
- **Supporting table 4**: Validation results and R2, calibrated range and determination limits of the calibration curves.
- **Supporting table 5**: FDR adjusted analytes contributing to the small differences between the study groups “caries active” (red), “caries inactive” (green) and “caries rehabilitated” (blue) in saliva in figure 1.
- **Supporting table 6**: FDR adjusted analytes contributing to the small differences between the study groups “caries active” (red), “caries inactive” (green) and “caries rehabilitated” (blue) in saliva in A) supporting figure 1 (only amino acids, organic acids and carbohydrates), and B) supporting figure 2 (only data from study perticipants with complete data sets (amino acids, organic acids, carbohydrates and fatty acids)).
- **Supporting table 7**: FDR adjusted analytes contributing to the small differences between the study groups “caries active” (red), “caries inactive” (green) and “caries rehabilitated” (blue) in pellicle in figure 2.
- **Supporting table 8**: Concentrations of stable isotope labeled amino acids in the IS-solution.
- **Supporting Figure 1**: A) The abundance of organic acids (green), amino acids (blue), carbohydrates (grey) and fatty acids (red) in saliva is comparable in the study groups. B) A clear separation of the study groups in the PCA based on targeted data (only amino acids, organic acids, carbohydrates) is not possible. C.1) A clear separation of the study groups in the PLS-DA based on targeted data (only amino acids, organic acids, carbohydrates) is not possible. C.2) Loadings plot of PLS-DA. D) Color-coded values (raw data were “glog-ed” for normalization) of the group mean concentrations (only amino acids, organic acids, carbohydrates) and box-whisker plots (whiskers show min – max, box shows 25%, 75% and median) of the saliva concentrations (nmol/mL) of the seven top-ranked compounds (ranking based on FDR adjusted ANOVA, no significant difference between groups were detected). Data are based on n=21 caries-active, n=18 caries-free and n=18 caries-rehabilitated/medically treated children. Missing values were KNN imputed. No fatty acid data included.
- **Supporting Figure 2**: A) The abundance of organic acids (green), amino acids (blue), carbohydrates (grey) and fatty acids (red) in saliva is comparable in the study groups. B) A clear separation of the study groups in the PCA based on targeted data (amino acids, organic acids, carbohydrates, fatty acids) is not possible. C.1) A clear separation of the study groups in the PLS-DA based on targeted data (only amino acids, organic acids, carbohydrates, fatty acids) is not possible. C.2) Loadings plot of PLS-DA. D) Color-coded values (raw data were “glog-ed” for normalization) of the group mean concentrations (amino acids, organic acids, carbohydrates, fatty acids) and box-whisker plots (whiskers show min – max, box shows 25%, 75% and median) of the saliva concentrations (nmol/mL) of the eight top-ranked compounds (ranking based on FDR adjusted ANOVA, no significant difference between groups were detected). Data are based on complete datasets n=11 caries-active, n=15 caries-free and n=12 caries-rehabilitated/medically treated children. Missing values were KNN imputed.
- **Supporting Figure 3:** Examples of LC-MS/MS(MRM)-chromatograms of amino acids and corresponding internal standards in pellicle (**A**), saliva (**B**) and standard (10 nmol/mL) (**C**). Labelling according to **Supporting Table 4**.
- **Supporting Figure 4:** Examples of LC-MS/MS(MRM)-chromatograms of organic acids and corresponding internal standards in pellicle (**A**), saliva (**B**) and standard (100 nmol/mL) (**C**). Labelling according to **Supporting Table 4**.
- **Supporting Figure 5:** Examples of LC-MS/MS(MRM)-chromatograms of carbohydrates and corresponding internal standards in pellicle (**A**), saliva (**B**), standard of monosaccharides and disaccharides (10 nmol/mL) (**C**) and standard of sugar-phosphates and oligosaccharides (**D**). Labelling according to **Supporting Table 4**.

**Supporting information**

**Supporting table 1**: Ionsource and path parameters for the detection of amino acids by UPLC-MS/MS in positive Electrospray (scheduled MRM).

| Q1 Mass (Da) | Q3 Mass (Da) | Retention Time (min) | ID | Window (sec) | DP (volts) | EP (volts) | CE (volts) | CXP (volts) |
| --- | --- | --- | --- | --- | --- | --- | --- | --- |
| 104.033 | 86.9, 85.9*, 68.9 | 3.5 | 4-Amino butyric acid | 30 | 101 | 10 | 15, 13, 21 | 10, 12, 10 |
| 132.053 | 132, 86* | 2.5 | L-Leucine | 30 | 116 | 10 | 7, 15 | 6, 10 |
| 150.027 | 103.9*, 56 | 3.1 | L-Methionine | 30 | 71 | 10 | 15, 23 | 12, 8 |
| 116.026 | 70*, 68 | 3.6 | L-Proline | 30 | 146 | 10 | 21, 39 | 10, 8 |
| 120.025 | 74*, 56 | 5.5 | L-Threonine | 30 | 146 | 10 | 15, 21 | 10, 8 |
| 147.045 | 83.9*, 130 | 6.6 | L-Glutamine | 30 | 46 | 10 | 23, 13 | 10, 6 |
| 94.023 | 76*, 32 | 4.9 | IS beta-Alanine (13C3, 15N) | 30 | 46 | 10 | 11, 23 | 10, 16 |
| 135.011 | 75*, 89 | 6.9 | IS Asparagine (15N2) | 30 | 66 | 10 | 21, 15 | 12, 10 |
| 139.01 | 76.9, 91.9* | 7.95 | IS Aspartic acid (13C4, 15N) | 30 | 46 | 10 | 19, 15 | 10, 12 |
| 154.024 | 88.9*, 107 | 7.1 | IS Glutamic acid (13C5, 15N) | 30 | 61 | 10 | 23, 15 | 12, 10 |
| 155.08 | 90*, 137.1 | 8.8 | IS Lysine (13C6, 15N2) | 30 | 51 | 10 | 23, 13 | 12, 6 |
| 171.089 | 125.1*, 106 | 2.4 | IS Phenylalanine (ring-d5) | 30 | 86 | 10 | 21, 39 | 6, 12 |
| 152.032 | 88*, 135 | 6.6 | IS Glutamine (13C5) | 30 | 46 | 10 | 25, 13 | 12, 6 |
| 153.026 | 107*, 56 | 3.1 | IS Methionine (Methyl-d3) | 30 | 51 | 10 | 15, 23 | 12, 8 |
| 162.038 | 115*, 87 | 8.3 | IS Histidine (13C6) | 60 | 61 | 10 | 21, 35 | 8, 10 |
| 181.031 | 73.9*, 121.1 | 8.6 | IS Arginine (13C6) | 30 | 61 | 10 | 29, 19 | 10, 6 |
| 186.028 | 140.1, 169 | 3.7 | IS Tyrosine (ring-d4) | 30 | 61 | 10 | 19, 13 | 8, 8 |
| 210.054 | 192.1*, 150 | 2.5 | IS Tryptophan (indole-d5) | 30 | 46 | 10 | 15, 25 | 8, 10 |
| 78.044 | 77.9, 32* | 5.8 | IS Glycine (2,2-d2) | 30 | 51 | 10 | 5, 17 | 10, 16 |
| 93.029 | 92.9, 46* | 5 | IS Alanine (13C3) | 30 | 41 | 10 | 5, 15 | 12, 8 |
| 109.004 | 61.9*, 44.1 | 6.74 | IS Serine (13C3) | 30 | 51 | 10 | 15, 31 | 8, 8 |
| 122.067 | 75*, 46 | 3.6 | IS Proline (13C5, 15N) | 30 | 56 | 10 | 23, 43 | 12, 8 |
| 124.043 | 77*, 59 | 3.4 | IS Valine (13C5, 15N) | 30 | 46 | 10 | 15, 29 | 10, 10 |
| 125.049 | 106, 79* | 2.5 | IS Threonine (13C4, 15N) | 30 | 136 | 10 | 25, 27 | 12, 12 |
| 134.014 | 87*, 45 | 2.5 | IS Leucine (1,2-13C2) | 30 | 51 | 10 | 15, 31 | 10, 8 |
| 138.08 | 91*, 74 | 2.8 | IS Isoleucine (13C6) | 30 | 51 | 10 | 15, 25 | 12, 10 |
| 156.051 | 110.1*, 83 | 8.3 | L-Histidine | 60 | 66 | 10 | 19, 33 | 8, 12 |
| 175.1 | 70*, 116 | 8.6 | L-Arginine | 30 | 71 | 10 | 31, 19 | 10, 6 |
| 133.007 | 73.9*, 87 | 6.9 | L-Asparagine | 30 | 56 | 10 | 21, 13 | 10, 12 |
| 148.024 | 83.9*, 102 | 7.1 | L-Glutamic acid | 30 | 41 | 10 | 23, 15 | 12, 12 |
| 182.03 | 136*, 90.9 | 3.7 | L-Tyrosine | 30 | 46 | 10 | 19, 39 | 6, 12 |
| 118.046 | 72*, 57 | 3.4 | L-Valine | 30 | 101 | 10 | 15, 39 | 10, 8 |
| 132.049 | 132, 86* | 2.8 | L-Isoleucine | 30 | 126 | 10 | 7, 15 | 6, 10 |
| 166.067 | 120*, 102.9 | 2.4 | L-Phenylalanine | 30 | 111 | 10 | 19, 37 | 6, 12 |
| 176.071 | 159*, 70 | 7.3 | Citrulline | 30 | 41 | 10 | 13, 31 | 10, 10 |
| 205.05 | 188*, 146 | 2.5 | L-Tryptophan | 30 | 51 | 10 | 15, 25 | 10, 10 |
| 133.993 | 73.9, 87.9* | 7.95 | L-Aspartic acid | 30 | 56 | 10 | 19, 15 | 10, 10 |
| 76.03 | 76, 30* | 5.8 | Glycine | 30 | 51 | 10 | 5, 17 | 10, 14 |
| 147.061 | 84*, 130 | 8.8 | L-Lysine | 30 | 56 | 10 | 23, 13 | 10, 6 |
| 129.999 | 130, 83.9* | 2.6 | L-Pyroglutamic acid | 30 | 66 | 10 | 9, 19 | 6, 10 |
| 106.017 | 59.9*, 42 | 6.74 | L-Serine | 30 | 51 | 10 | 15, 29 | 8, 8 |
| 170.041 | 124*, 96, 82.9 | 7.4 | 1-Methylhistidine | 60 | 61 | 10 | 21, 31, 33 | 6, 12, 10 |
| 104.042 | 86*, 57, 59 | 3.8 | 3-Aminoisobutyric acid | 30 | 51 | 10 | 11, 19, 33 | 12, 8, 10 |
| 170.046 | 96*, 95.1, 109.1 | 8.4 | 3-Methylhistidine | 30 | 66 | 10 | 27, 41, 21 | 12, 8, 8 |
| 162.026 | 97.9*, 116 | 6.3 | Aminoadipic acid | 30 | 51 | 10 | 21, 17 | 12, 14 |
| 90.006 | 89.9, 71.9* | 4.9 | beta-Alanine | 30 | 46 | 10 | 7, 11 | 12, 10 |
| 131.952 | 132, 89.9* | 5.1 | Creatine | 30 | 161 | 10 | 9, 17 | 6, 12 |
| 131.931 | 132, 86.1* | 5.1 | 4-Hydroxyproline | 30 | 101 | 10 | 7, 19 | 6, 6 |
| 89.888 | 44.1*, 45.1 | 5 | L-Alanine | 30 | 1 | 10 | 13, 41 | 8, 10 |
| 119.907 | 74*, 56 | 5.8 | L-Homoserine | 30 | 21 | 10 | 15, 23 | 10, 8 |
| 89.947 | 44*, 30 | 4.4 | Sarcosine | 30 | 31 | 10 | 15, 55 | 6, 6 |

*Quantifier-transition

**Supporting table 2**: Ionsource and path parameters for the detection of derivatized organic acids by UPLC-MS/MS in negative Electrospray.

| Q1 Mass (Da) | Q3 Mass (Da) | Dwell Time (msec) | ID | DP (volts) | EP (volts) | CE (volts) | CXP (volts) |
| --- | --- | --- | --- | --- | --- | --- | --- |
| 209.987 | 136.8*, 151.8 | 5 | Glycolic acid 3NPH | -10 | -10 | -20, -20 | -21, -17 |
| 224.012 | 152.1*, 137.9 | 5 | L(+) Lactic acid 3NPH | -20 | -10 | -20, -20 | -19, -17 |
| 353.083 | 205.9*, 222.9, 146 | 5 | D-Pantothenic acid1 3NPH | -125 | -10 | -26, -22, -28 | -25, -25, -7 |
| 270.034 | 137*, 210.1, 151.9 | 5 | Threonic acid 3NPH | -60 | -10 | -28, -20, -22 | -15, -19, -17 |
| 195.961 | 151.9*, 151 | 5 | Sodium Acetate (13C2) 3NPH | -65 | -10 | -18, -20 | -17, -11 |
| 209.573 | 136.9*, 167 | 5 | Propionic acid (2,2-D2) 3NPH | -120 | -10 | -28, -20 | -7, -3 |
| 193.915 | 151.7*, 150.9 | 5 | Acetic acid 3NPH | -40 | -10 | -20, -18 | -37, -1 |
| 207.929 | 136.8, 164.9* | 5 | Propionic acid 3NPH | -65 | -10 | -28, -18 | -9, -13 |
| 211.925 | 138*, 152 | 5 | Glycolic acid (2,2-D2) 3NPH | -75 | -10 | -20, -20 | -19, -19 |
| 227.035 | 151.9*, 137 | 5 | Lactic acid (13C3) 3NPH | -60 | -10 | -22, -26 | -19, -19 |
| 221.956 | 136.9, 151.9* | 5 | Butyric acid 3NPH | -105 | -10 | -24, -20 | -37, -23 |
| 236.079 | 136.8, 152* | 5 | 2-Methylbutyrate 3NPH | -95 | -10 | -26, -22 | -15, -5 |
| 250.007 | 136.7* | 5 | Hexanoic acid 3NPH | -25 | -10 | -34 | -11 |
| 222.037 | 136.9, 178.9*, 106.9 | 5 | Isobutyrate 3NPH | -85 | -10 | -28, -18, -34 | -17, -15, -11 |
| 236.019 | 137, 151.8* | 5 | Isovalerate 3NPH | -110 | -10 | -24, -22 | -1, -21 |
| 236.039 | 136.8, 151.9* | 5 | Pentanoic acid 3NPH | -95 | -10 | -30, -24 | -15, -17 |
| 250.066 | 136.9*, 152.2 | 5 | 4-Methylvaleric acid 3NPH | -95 | -10 | -28, -24 | -11, -21 |
| 224.996 | 137*, 152 | 5 | Butyrate (13C4) 3NPH | -100 | -10 | -26, -22 | -15, -17 |
| 357.009 | 136.9, 204* | 5 | Pyruvic acid2 3NPH | -80 | -10 | -30, -24 | -19, -3 |
| 371.082 | 150, 178*, 137 | 5 | 2-Ketobutyric acid1 3NPH | -95 | -10 | -26, -24, -30 | -11, -21, -11 |
| 577.989 | 383*, 427, 245.9 | 5 | cis-Aconitic acid3 3NPH | -165 | -10 | -28, -24, -36 | -13, -15, -29 |
| 385.01 | 232, 212.9* | 5 | Fumaric acid2 3NPH | -145 | -10 | -24, -22 | -27, -9 |
| 401.057 | 247.9*, 152 | 5 | Glutaric acid2 3NPH | -170 | -10 | -26, -30 | -25, -11 |
| 358.991 | 149.9*, 178 | 5 | Oxalic acid2 3NPH | -105 | -10 | -26, -24 | -15, -21 |
| 387.092 | 234, 97.9* | 5 | Succinat2 3NPH | -125 | -10 | -24, -44 | -27, -11 |
| 596.004 | 221.9*, 401 | 5 | Citric acid3 3NPH | -210 | -10 | -38, -26 | -19, -51 |
| 358.983 | 137, 206* | 5 | Pyruvat2 (13C2) 3NPH | -10 | -10 | -30, -22 | -7, -31 |
| 386.926 | 236, 234* | 5 | Fumaric acid2 (2,3-D2) 3NPH | -110 | -10 | -20, -24 | -11, -15 |
| 404.974 | 251*, 249.8 | 5 | Glutaric acid2 (2,2,4,4-D4) 3NPH | -175 | -10 | -26, -26 | -27, -21 |
| 373.914 | 177.9*, 149.9 | 5 | Malonic acid2 (2-13C) 3NPH | -110 | -10 | -22, -30 | -19, -11 |
| 372.887 | 177.9*, 149.8 | 5 | Malonic acid2 3NPH | -95 | -10 | -22, -30 | -11, -7 |
| 360.929 | 151*, 178.8 | 5 | Oxalic acid2 (1,2-13C2) 3NPH | -110 | -10 | -28, -24 | -13, -17 |
| 390.972 | 237*, 236 | 5 | Succinic acid2 (D4) 3NPH | -120 | -10 | -26, -26 | -17, -19 |
| 598.047 | 221.9*, 137, 247.1 | 5 | Citric acid3 (13C2) 3NPH | -80 | -10 | -40, -70, -38 | -13, -7, -5 |

*Quantifier-transition

**Supporting table 3:** Ionsource and path parameters for the detection of derivatized carbohydrates by UPLC-MS/MS in negative Electrospray.

| Q1 Mass (Da) | Q3 Mass (Da) | Dwell Time (msec) | ID | DP (volts) | EP (volts) | CE (volts) | CXP (volts) |
| --- | --- | --- | --- | --- | --- | --- | --- |
| 283.883 | 160.1*, 92.1, 131.8 | 20 | Fucose | -105 | -10 | -24, -54, -30 | -11, -5, -7 |
| 299.909 | 160, 58.9*, 91.8 | 20 | Galactose (+Mannose) | -85 | -10 | -28, -48, -50 | -5, -9, -11 |
| 306.102 | 164, 60.8*, 92 | 20 | Glucose 13C6 | -55 | -10 | -28, -46, -30 | -5, -7, -5 |
| 379.924 | 78.9*, 96.8, 282.1 | 20 | Glucose-6-phosphate | -50 | -10 | -64, -56, -28 | -7, -9, -5 |
| 461.999 | 58.9*, 282, 70.9 | 20 | Lactose | -65 | -10 | -78, -36, -66 | -7, -9, -9 |
| 269.676 | 91.9, 160, 70.9* | 20 | Ribose | -60 | -10 | -34, -24, -28 | -5, -5, -7 |
| 274.841 | 92, 74*, 163.9 | 20 | Ribose 13C5 | -50 | -10 | -34, -28, -24 | -5, -9, -7 |
| 349.895 | 78.7*, 97, 252 | 20 | Ribose-5-phosphate | -80 | -10 | -76, -32, -26 | -9, -5, -5 |
| 299.91 | 160.001*, 58.901, 91.801 | 20 | Glucose | -85 | -10 | -28, -48, -50 | -5, -9, -11 |
| 461.998 | 58.9*, 282, 70.9 | 20 | Isomaltose | -65 | -10 | -78, -36, -66 | -7, -9, -9 |
| 269.677 | 91.9, 160*, 70.9 | 20 | Xylose | -60 | -10 | -34, -24, -28 | -5, -5, -7 |
| 461.997 | 58.9*, 282, 70.9 | 20 | Maltose | -65 | -10 | -78, -36, -66 | -7, -9, -9 |
| 624.084 | 220.9*, 220.8, 58.9 | 20 | Maltotriose | -10 | -10 | -54, -54, -110 | -3, -15, -5 |
| 786.168 | 58.9*, 220.9, 282 | 20 | Maltotetraose | -15 | -10 | -124, -66, -60 | -9, -7, -3 |
| 948.211 | 221.1*, 383, 282.1 | 20 | Maltopentaose | -195 | -10 | -82, -76, -72 | -7, -9, -3 |
| 1110.224 | 220.8, 383*, 221 | 20 | Maltohexaose | -35 | -10 | -88, -90, -92 | -27, -5, -25 |
| 635.698 | 58.8, 1092.3*, 930.2 | 20 | Maltoheptaose | -150 | -10 | -114, -36, -38 | -7, -9, -5 |

*Quantifier-transition

**Supporting table 4**: Validation results and R^2^, calibrated range and determination limits of the calibration curves.

|  | **Nominal concentration (nmol/mL)** | | | |  | |  | |  | |  | |  | |
| --- | --- | --- | --- | --- | --- | --- | --- | --- | --- | --- | --- | --- | --- | --- |
| **Analyte** | **1** | **10** | **100** |  | |  | |  | |  | |  | |  |
|  | **Found concentration±SD (RSD), recovery** | | | | **RSD (%)** | | **r^2^** | | **Calibrated range [nmol/mL]** | | **LLOQ [nmol/mL]** | | **LOD [nmol/mL]** | |
| L-Leucine (I-1) | 0.799±0.087 (10.84), 79.89% | 9.439±0.337 (3.57), 94.39% | 93.869±1.574 (1.68), 93.87% | 1.12 | | 0.999 | | 0.05-100 | | 0.05 | | 0.025 | |  |
| L-Methionine (I-2) | 0.920±0.062 (6.73), 91.97% | 9.863±0.415 (4.20), 98.63% | 91.452±1.150 (1.26), 91.45% | 0.99 | | 0.993 | | 0.025-100 | | 0.025 | | 0.005 | |  |
| L-Proline (I-3) | 0.859±0.049 (5.76), 85.86% | 9.740±0.278 (2.86), 97.40% | 90.390±1.831 (2.03), 90.39% | 2.96 | | 0.995 | | 0.1-100 | | 0.1 | | 0.05 | |  |
| L-Threonine (I-4) | 0.749±0.167 (22.23), 74.92% | 7.738±0.236 (3.05), 77.38% | 121.579±4.204 (3.46), 121.58% | 2.66 | | 0.998 | | 1-100 | | 1 | | 0.5 | |  |
| L-Glutamine (I-5) | 0.847±0.053 (6.21), 84.66% | 9.130±0.301 (3.29), 91.30% | 106.641±4.833 (4.53), 106.64% | 0.92 | | 0.999 | | 0.025-100 | | 0.025 | | 0.025 | |  |
| L-Histidine (I-6) | 0.892±0.025 (2.79), 89.24% | 9.428±0.311 (3.30), 94.28% | 101.911±1.906 (1.87), 101.91% | 0.72 | | 0.999 | | 0.05-100 | | 0.05 | | 0.01 | |  |
| L-Arginine (I-7) | 0.727±0.099 (13.68), 72.69% | 8.983±0.304 (3.38), 89.83% | 99.044±1.539 (1.55), 99.04% | 1.87 | | 0.999 | | 0.1-100 | | 0.1 | | 0.005 | |  |
| L-Glutamic acid (I-8) | 0.720±0.088 (12.25), 72.00% | 9.059±0.338 (3.73), 90.59% | 98.187±2.050 (2.09), 98.19% | 1.7 | | 0.999 | | 0.05-100 | | 0.05 | | 0.025 | |  |
| L-Tyrosine (I-9) | 0.782±0.057 (7.35), 78.17% | 8.487±0.203 (2.39), 84.87% | 89.581±2.290 (2.56), 89.58% | 1.55 | | 0.999 | | 0.025-100 | | 0.025 | | 0.01 | |  |
| L-Valine (I-10) | 0.805±0.079 (9.82), 80.46% | 9.491±0.383 (4.04), 94.91% | 98.383±1.279 (1.30), 98.38% | 1.07 | | 0.999 | | 0.05-100 | | 0.05 | | 0.025 | |  |
| L-Isoleucine (I-11) | 0.812±0.037 (4.60), 81.24% | 8.912±0.365 (4.09), 89.12% | 95.019±1.573 (1.66), 95.02% | 1.53 | | 0.999 | | 0.05-100 | | 0.05 | | 0.025 | |  |
| L-Phenylalanine (I-12) | 0.854±0.100 (11.76), 85.36% | 9.512±0.400 (4.21), 95.12% | 93.828±1.371 (1.46), 93.83% | 0.71 | | 0.996 | | 0.05-100 | | 0.05 | | 0.01 | |  |
| Citrulline (I-13) | 1.046±0.060 (5.72), 104.58% | 10.570±0.420 (3.98), 105.70% | 97.513±1.256 (1.29), 97.51% | 2.14 | | 0.999 | | 0.025-100 | | 0.025 | | 0.01 | |  |
| L-Tryptophan (I-14) | 0.977±0.044 (4.55), 97.72% | 10.084±0.585 (5.81), 100.84% | 105.418±2.158 (2.05), 105.42% | 7.81 | | 0.999 | | 0.01-100 | | 0.01 | | 0.005 | |  |
| L-Aspartic acid (I-15) | 0.747±0.048 (6.36), 74.65% | 8.607±0.336 (3.91), 86.07% | 92.852±1.275 (1.37), 92.85% | 1.93 | | 0.999 | | 0.25-100 | | 0.25 | | 0.05 | |  |
| L-Lysine (I-16) | 0.755±0.065 (8.54), 75.51% | 9.167±0.257 (2.80), 91.67% | 97.259±1.407 (1.45), 97.26% | 1.07 | | 0.999 | | 0.25-100 | | 0.25 | | 0.1 | |  |
| L-Pyroglutamic acid (I-17) | 0.947±0.055 (5.79), 94.71% | 9.087±0.220 (2.42), 90.87% | 103.283±3.241 (3.14), 103.28% | 3.32 | | 0.997 | | 0.1-100 | | 0.1 | | 0.01 | |  |
| L-Serine (I-18) | 0.589±0.057 (9.70), 58.87% | 9.282±0.532 (5.73), 92.82% | 102.271±0.848 (0.83), 102.27% | 1.88 | | 0.998 | | 0.25-100 | | 0.25 | | 0.1 | |  |
| 1-Methylhistidine (I-19) | 0.849±0.027 (3.14), 84.91% | 8.957±0.208 (2.33), 89.57% | 74.639±1.643 (2.20), 74.64% | 2.54 | | 0.998 | | 0.1-100 | | 0.1 | | 0.01 | |  |
| 3-Aminoisobutyric acid (I-20) | 1.452±0.085 (5.88), 145.24% | 14.143±0.366 (2.59), 141.43% | 153.592±4.430 (2.88), 153.59% | 5.92 | | 0.999 | | 0.05-100 | | 0.05 | | 0.025 | |  |
| 3-Methylhistidine (I-21) | 0.941±0.026 (2.77), 94.05% | 9.519±0.321 (3.37), 95.19% | 89.838±1.322 (1.47), 89.84% | 1.57 | | 0.998 | | 0.025-100 | | 0.025 | | 0.01 | |  |
| 4-Aminobutyric acid (I-22) | / | 0.549±0.011 (2.05), 5.49% | 4.358±0.300 (6.89), 4.36% | 19.56 | | 0.998 | | 0.25-100 | | 0.25 | | 0.25 | |  |
| Aminoadipic acid (I-23) | 1.052±0.049 (4.61), 105.23% | 10.904±0.335 (3.07), 109.04% | 123.217±1.975 (1.60), 123.22% | 1.13 | | 0.999 | | 0.01-100 | | 0.01 | | 0.005 | |  |
| beta-Alanine (I-24) | 0.892±0.036 (3.99), 89.15% | 9.164±0.270 (2.95), 91.64% | 99.868±2.079 (2.08), 99.87% | 1.18 | | 0.999 | | 0.25-100 | | 0.25 | | 0.1 | |  |
| Creatine (I-25) | 0.891±0.040 (4.47), 89.13% | 10.075±0.422 (4.19), 100.75% | 98.319±1.734 (1.76), 98.32% | 4.07 | | 0.998 | | 0.05-100 | | 0.05 | | 0.05 | |  |
| 4-Hydroxyproline (I-26) | 0.911±0.027 (2.98), 91.13% | 9.143±0.321 (3.51), 91.43% | 97.465±1.854 (1.90), 97.46% | 2.28 | | 0.998 | | 0.1-100 | | 0.1 | | 0.05 | |  |
| L-Alanine (I-27) | 0.482±0.106 (22.01), 48.18% | 9.321±0.517 (5.54), 93.21% | 92.593±1.116 (1.21), 92.59% | 2.39 | | 0.997 | | 0.25-100 | | 0.25 | | 0.05 | |  |
| Sarcosine (I-28) | 0.830±0.017 (2.09), 82.95% | 8.735±0.183 (2.09), 87.35% | 100.254±2.167 (2.16), 100.25% | 1.52 | | 0.999 | | 0.025-100 | | 0.025 | | 0.01 | |  |
| L-Asparagine (I-29) | 0.917±0.099 (10.81), 91.68% | 9.601±0.348 (3.62), 96.01% | 91.839±2.297 (2.50), 91.84% | 1.75 | | 0.998 | | 0.25-100 | | 0.25 | | 0.1 | |  |
| Glycine (I-30) | < LLOQ | 9.575±0.458 (4.79), 95.75% | 95.244±3.583 (3.76), 95.24% | 2.04 | | 0.996 | | 2.5-100 | | 2.5 | | 1 | |  |
| L-Homoserine (I-31) | 0.863±0.062 (7.18), 86.35% | 9.684±0.252 (2.60), 96.84% | 94.379±3.354 (3.55), 94.38% | 2.59 | | 0.997 | | 0.1-100 | | 0.1 | | 0.025 | |  |
| Glycolic acid (II-1) | 0.57±0.05 (9.26), 56.6% | 7.05±0.34 (4.85), 70.5% | 70.68±0.38 (0.54), 70.7% | 7.25 | | 0.989 | | 0.98-1000 | | 0.98 | | 0.12 | |  |
| L(+) Lactic acid (II-2) | 7.73±11.82 (152.93), 772.8% | 19.88±17.08 (85.94), 198.8% | 57.27±1.73 (3.03), 57.3% | 7.63 | | 0.972 | | 0.98-1000 | | 0.98 | | 0.12 | |  |
| D-Pantothenic acid (II-3) | 0.35±0.01 (2.42), 34.5% | 4.51±1.08 (23.98), 45.1% | 49.84±0.90 (1.81), 49.8% | 12.91 | | 0.995 | | 0.24-500 | | 0.24 | | 0.12 | |  |
| Threonic acid (II-4) | 0.33±0.02 (5.37), 33.2% | 3.86±1.12 (29.13), 38.6% | 32.52±0.70 (2.17), 32.5% | 15.60 | | 0.996 | | 0.49-1000 | | 0.49 | | 0.12 | |  |
| Acetic acid (II-5) | < LLOQ | 8.48±3.65 (43.05), 84.8% | 62.55±1.30 (2.08), 62.5% | 4.31 | | 0.993 | | 3.9-1000 | | 3.9 | | 0.12 | |  |
| Propionic acid (II-6) | 0.74±0.03 (4.35), 74.5% | 8.05±1.68 (20.85), 80.5% | 64.41±1.62 (2.52), 64.4% | 8.41 | | 0.988 | | 0.49-1000 | | 0.49 | | 0.12 | |  |
| Butyric acid (II-7) | 0.62±0.05 (8.60), 62.5% | 7.79±0.76 (9.72), 77.9% | 75.10±4.30 (5.72), 75.1% | 10.04 | | 0.994 | | 0.98-1000 | | 0.98 | | 0.12 | |  |
| 2-Methylbutyrate (II-8) | 0.91±0.06 (7.06), 90.9% | 8.69±0.53 (6.15), 86.9% | 87.06±4.39 (5.04), 87.1% | 10.07 | | 0.995 | | 0.98-1000 | | 0.98 | | 0.12 | |  |
| Hexanoic acid (II-9) | 0.53±0.12 (22.32), 53.0% | 8.82±1.27 (14.44), 88.2% | 71.68±2.06 (2.87), 71.7% | 10.31 | | 0.996 | | 0.24-500 | | 0.24 | | 0.12 | |  |
| Isobutyrate (II-10) | 0.66±0.04 (5.71), 65.7% | 7.77±0.50 (6.40), 77.7% | 74.81±7.74 (9.98), 74.8% | 10.33 | | 0.983 | | 0.12-1000 | | 0.12 | | 0.12 | |  |
| Isovalerate (II-11) | 0.69±0.03 (5.02), 69.4% | 7.68±0.48 (6.29), 76.8% | 74.82±1.30 (1.74), 74.8% | 10.61 | | 0.994 | | 0.24-500 | | 0.24 | | 0.12 | |  |
| Pentanoic acid (II-12) | 0.74±0.05 (6.66), 73.7% | 7.76±0.88 (11.38), 77.6% | 71.92±2.79 (3.89), 71.9% | 8.37 | | 0.993 | | 0.98-1000 | | 0.98 | | 0.12 | |  |
| 4-Methylvaleric acid (II-13) | 0.68±0.01 (0.95), 67.8% | 7.69±0.60 (7.68), 76.9% | 67.25±1.01 (1.51), 67.3% | 9.04 | | 0.994 | | 0.12-250 | | 0.12 | | 0.12 | |  |
| Pyruvic acid (II-14) | 0.63±0.34 (53.76), 62.5% | 6.77±0.18 (2.70), 67.7% | 66.62±0.91 (1.36), 66.6% | 3.22 | | 0.994 | | 0.98-1000 | | 0.98 | | 0.12 | |  |
| 2-Ketobutyric acid (II-15) | 0.32±0.02 (5.00), 32.3% | 4.25±0.09 (2.11), 42.5% | 34.93±0.61 (1.75), 34.9% | 3.51 | | 0.976 | | 0.98-500 | | 0.98 | | 0.12 | |  |
| cis-Aconitic acid (II-16) | 2.32±0.07 (3.05), 231.9% | 20.57±9.65 (46.94), 205.7% | 251.24±4.17 (1.66), 251.2% | 6.67 | | 0.994 | | 0.98-125 | | 0.98 | | 0.12 | |  |
| Fumaric acid (II-17) | < LLOQ | 2.33±1.38 (59.02), 23.3% | 152.44±5.34 (3.51), 152.4% | 8.65 | | 0.966 | | 3.9-500 | | 3.9 | | 0.12 | |  |
| Glutaric acid (II-18) | 0.53±0.01 (1.59), 52.9% | 6.97±0.13 (1.84), 69.7% | 69.61±0.51 (0.73), 69.6% | 4.55 | | 0.992 | | 0.98-1000 | | 0.98 | | 0.12 | |  |
| Oxalic acid (II-19) | < LLOQ | 6.21±0.22 (3.51), 62.1% | 33.57±8.65 (25.77), 33.6% | 45.01 | | 0.995 | | 7.8-1000 | | 7.8 | | 0.12 | |  |
| Succinat (II-20) | 0.56±0.02 (3.57), 55.9% | 6.19±0.09 (1.42), 61.9% | 64.63±0.85 (1.32), 64.6% | 2.44 | | 0.996 | | 0.49-1000 | | 0.49 | | 0.12 | |  |
| Citric acid (II-21) | < LLOQ | 7.89±0.31 (3.93), 78.9% | 74.98±1.75 (2.34), 75.0% | 11.46 | | 0.995 | | 3.91-1000 | | 3.91 | | 0.98 | |  |
| Malonic acid (II-22) | 0.49±0.01 (1.66), 49.5% | 6.00±1.16 (19.36), 60.0% | 53.68±1.36 (2.53), 53.7% | 6.24 | | 0.998 | | 0.49-500 | | 0.49 | | 0.12 | |  |
| Fucose (III-1) | 1.16±0.08 (6.70), 116.45% | 9.03±0.16 (1.82), 90.25% | 112.68±1.98 (1.76), 112.68% | 3.50 | | 0.998 | | 0.01-500 | | 0.01 | | 0.001 | |  |
| Lactose (III-2) | 1.04±0.02 (1.89), 104.12% | 9.65±0.75 (7.82), 96.47% | 90.24±0.97 (1.08), 90.24% | 10.92 | | 0.996 | | 0.01-50 | | 0.01 | | 0.001 | |  |
| Ribose (III-3) | 0.98±0.07 (6.83), 97.73% | 8.78±0.05 (0.53), 87.83% | 91.33±1.80 (1.97), 91.33% | 2.93 | | 0.998 | | 0.01-500 | | 0.01 | | 0.001 | |  |
| Glucose (III-4) | 1.06±0.04 (3.98), 106.39% | 9.50±0.27 (2.82), 94.97% | 98.82±1.14 (1.15), 98.82% | 3.41 | | 0.998 | | 0.1-500 | | 0.1 | | 0.001 | |  |
| Isomaltose (III-5) | 0.97±0.05 (4.66), 97.36% | 9.03±0.19 (2.15), 90.26% | 96.70±1.53 (1.58), 96.70% | 7.79 | | 0.995 | | 0.1-100 | | 0.1 | | 0.001 | |  |
| Xylose (III-6) | 0.91±0.08 (8.35), 90.94% | 8.94±0.09 (1.01), 89.40% | 93.48±2.00 (2.14), 93.48% | 3.62 | | 0.999 | | 0.01-500 | | 0.01 | | 0.001 | |  |
| Maltose (III-7) | 0.95±0.03 (2.77), 94.54% | 8.95±0.17 (1.86), 89.49% | 85.35±2.27 (2.66), 85.35% | 1.69 | | 1.000 | | 0.05-500 | | 0.05 | | 0.001 | |  |
| Glucose-6-phosphate (III-8) | 1.27±0.09 (7.46), 126.64% | 12.75±1.30 (10.17), 127.48% | 92.97±3.93 (4.23), 92.97% | 10.34 | | 0.991 | | 0.1-50 | | 0.1 | | 0.001 | |  |
| Ribose-5-phosphate (III-9) | 0.88±0.05 (5.90), 88.14% | 10.28±0.51 (4.96), 102.84% | 78.03±5.61 (7.19), 78.03% | 1.89 | | 0.996 | | 0.05-50 | | 0.05 | | 0.001 | |  |
| Maltotriose (III-10) | 1.13±0.07 (6.25), 113.01% | 10.95±0.58 (5.28), 109.49% | 89.37±4.80 (5.38), 89.37% | 1.96 | | 0.997 | | 0.05-50 | | 0.05 | | 0.005 | |  |
| Maltotetraose (III-11) | 0.96±0.06 (6.67), 96.40% | 9.93±0.85 (8.56), 99.34% | 89.32±4.33 (4.85), 89.32% | 3.84 | | 0.996 | | 0.05-100 | | 0.05 | | 0.005 | |  |
| Maltopentaose (III-12) | 0.85±0.06 (6.69), 85.27% | 8.62±0.58 (6.70), 86.18% | 72.63±3.50 (4.81), 72.63% | 3.90 | | 0.994 | | 0.05-100 | | 0.05 | | 0.01 | |  |
| Maltohexaose (III-13) | 0.76±0.06 (7.43), 75.86% | 8.76±0.67 (7.62), 87.64% | 82.13±4.36 (5.31), 82.13% | 3.30 | | 0.998 | | 0.13-130 | | 0.13 | | 0.065 | |  |
| Maltoheptaose (III-14) | 0.39±0.06 (14.04), 39.30% | 5.00±0.87 (17.46), 50.04% | 43.07±1.73 (4.02), 43.07% | 14.72 | | 0.993 | | 0.5-50 | | 0.5 | | 0.05 | |  |
|  | **2** | **20** | **200** |  | |  | |  | |  | |  | |  |
|  | **Found concentration±SD (RSD), recovery** | | |  | |  | |  | |  | |  | |  |
| Galactose+Mannose (III-15) | 1.90±0.16 (8.39), 94.83% | 17.88±0.22 (1.25), 89.41% | 214.26±2.05 (0.96), 107.13% | 2.70 | | 0.994 | | 0.2-1000 | | 0.2 | | 0.002 | |  |

**Supporting table 5:** FDR adjusted analytes contributing to the small differences between the study groups “caries active” (red), “caries inactive” (green) and “caries rehabilitated” (blue) in saliva in **figure 1**.

|  |  |  |  |  |  |  |
| --- | --- | --- | --- | --- | --- | --- |
| **Name** | **f.value** | **p.value** | **-log10(p)** | **FDR** | **Post-hoc tests** |  |
| Oleate | 6.4522 | 0.0030716 | 2.5126 | 0.16827 | free - active; rehabilitated - active | 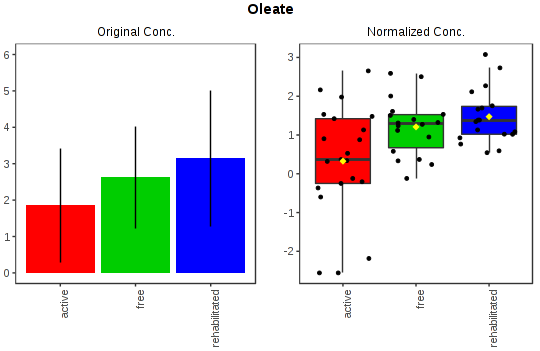 |
| L-Serine | 5.3744 | 0.0074368 | 2.1286 | 0.16827 | active - free; rehabilitated - free | 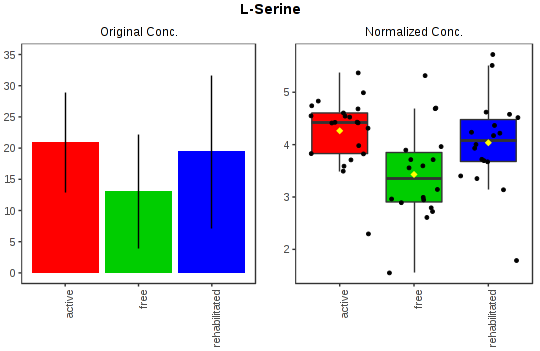 |
| L-Pyroglutamate | 4.6903 | 0.013237 | 1.8782 | 0.16827 | active - free; rehabilitated - free | 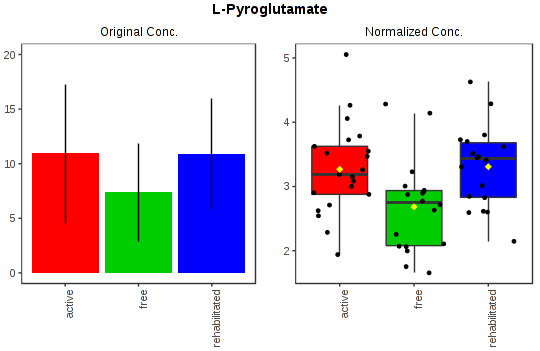 |
| 4-Hydroxyproline | 4.6357 | 0.013869 | 1.858 | 0.16827 | rehabilitated - active; rehabilitated - free | 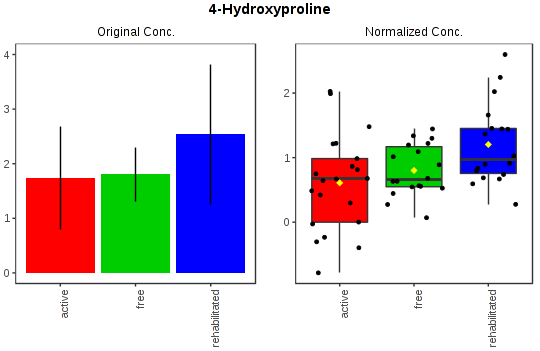 |
| L-Threonine | 4.4629 | 0.016079 | 1.7937 | 0.16827 | active - free; rehabilitated - free | 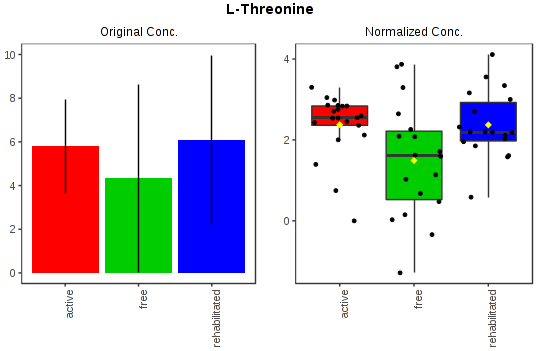 |
| 1-Methylhistidine | 4.4292 | 0.016551 | 1.7812 | 0.16827 | rehabilitated - active; rehabilitated - free | 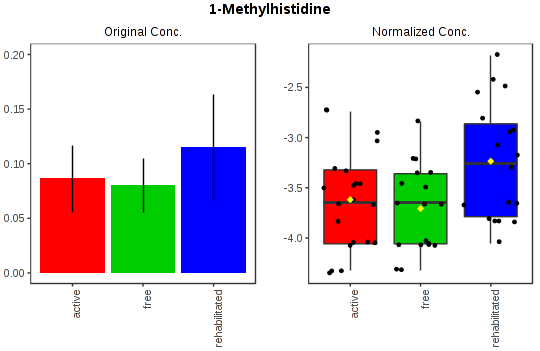 |
| Linoleate | 4.1341 | 0.021353 | 1.6705 | 0.17321 | rehabilitated - active; rehabilitated - free | 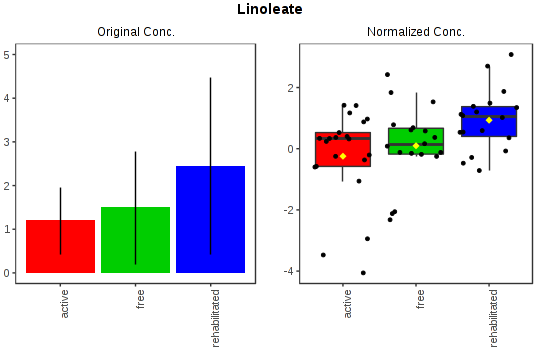 |
| Lactate | 3.9118 | 0.025911 | 1.5865 | 0.17321 | active - free; rehabilitated - free | 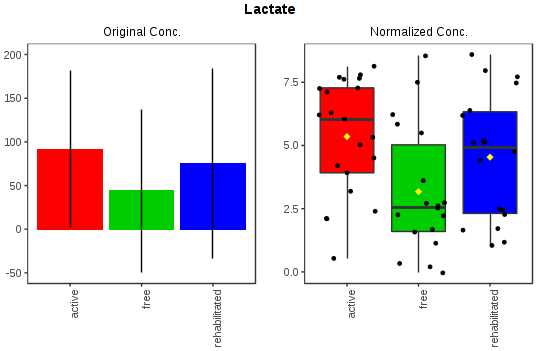 |
| Glucose | 3.737 | 0.030196 | 1.5201 | 0.17321 | active - free; rehabilitated - free | 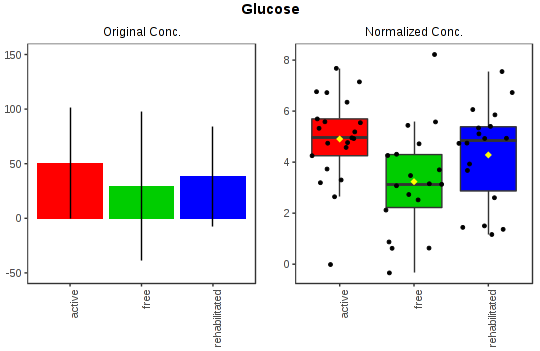 |
| Palmitoleate | 3.7299 | 0.030385 | 1.5173 | 0.17321 | free - active; rehabilitated - active | 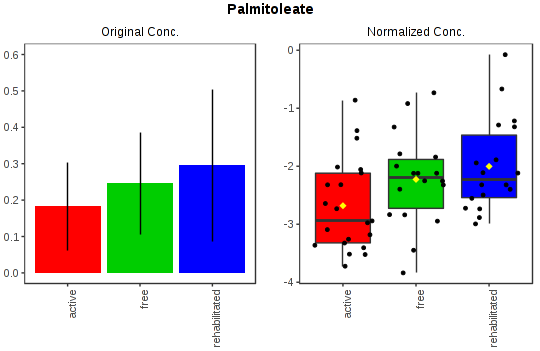 |
| Threonate | 3.6985 | 0.031235 | 1.5054 | 0.17321 | active - free; rehabilitated - free | 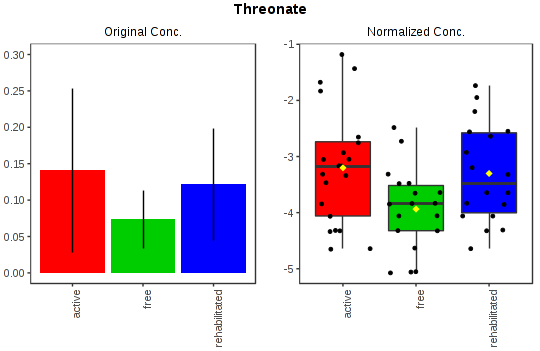 |
| Succinate | 3.493 | 0.037448 | 1.4266 | 0.19036 | active - free; active - rehabilitated | 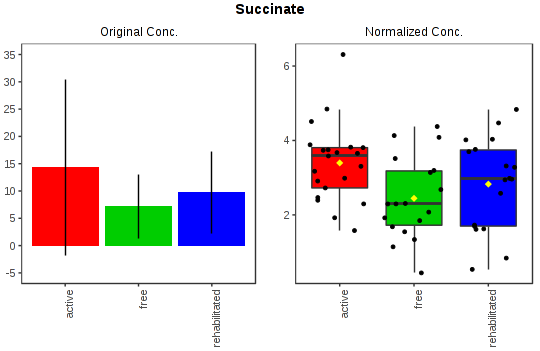 |

**Supporting table 6:** FDR adjusted analytes contributing to the small differences between the study groups “caries active” (red), “caries inactive” (green) and “caries rehabilitated” (blue) in saliva in **A)** **supporting** **figure 1** (only amino acids, organic acids and carbohydrates), and **B) supporting figure 2** (only data from study participants with complete data sets (amino acids, organic acids, carbohydrates and fatty acids)).

|  |  |  |  |  |  |  |  |
| --- | --- | --- | --- | --- | --- | --- | --- |
| **A** | **Name** | **f.value** | **p.value** | **-log10(p)** | **FDR** | **Post-hoc tests** |  |
|  | L-Serine | 5.3744 | 0.0074368 | 2.1286 | 0.14344 | active - free; rehabilitated - free | 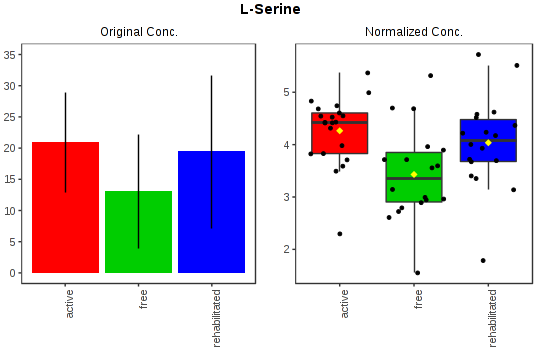 |
|  | L-Pyroglutamate | 4.6903 | 0.013237 | 1.8782 | 0.14344 | active - free; rehabilitated - free | 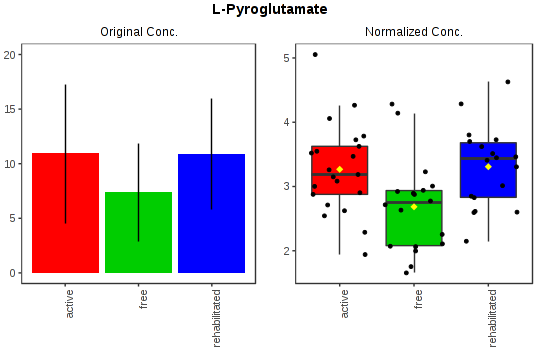 |
|  | 4-Hydroxyproline | 4.6357 | 0.013869 | 1.858 | 0.14344 | rehabilitated - active; rehabilitated - free | 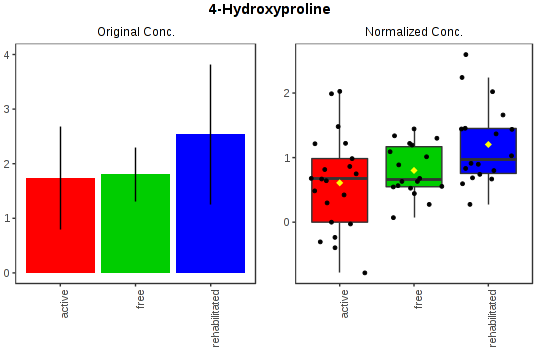 |
|  | Glucose | 4.5659 | 0.01472 | 1.8321 | 0.14344 | active - free; rehabilitated - free | 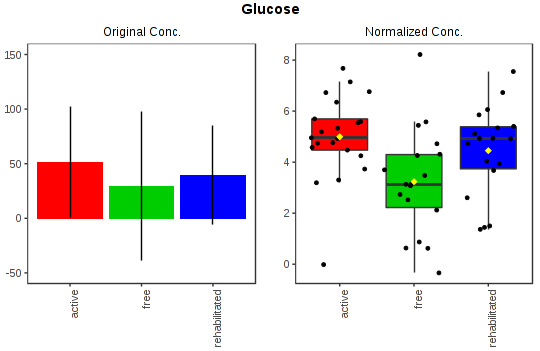 |
|  | L-Threonine | 4.4629 | 0.016079 | 1.7937 | 0.14344 | active - free; rehabilitated - free | 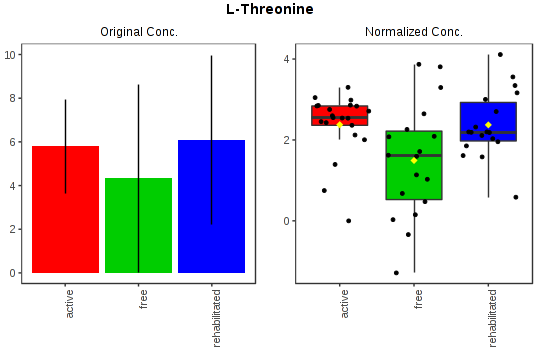 |
|  | 1-Methylhistidine | 4.4292 | 0.016551 | 1.7812 | 0.14344 | rehabilitated - active; rehabilitated - free | 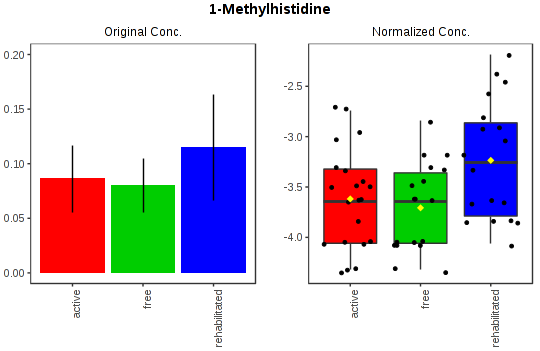 |
|  | Pyruvate | 4.0532 | 0.022906 | 1.64 | 0.17016 | active - free; active - rehabilitated | 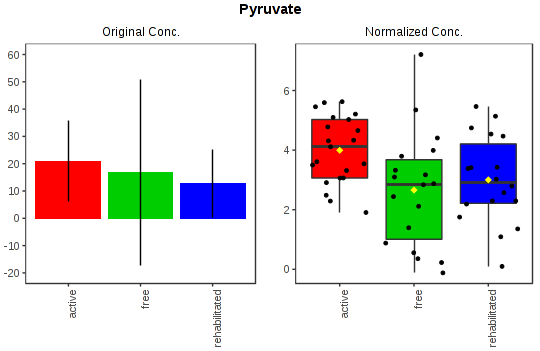 |
| **B** | **Name** | **f.value** | **p.value** | **-log10(p)** | **FDR** | **Post-hoc tests** |  |
|  | Pyruvate | 7.2076 | 0.0023912 | 2.6214 | 0.13838 | active - free; active - rehabilitated | 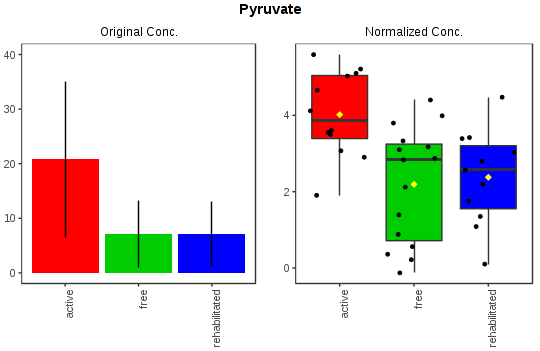 |
|  | Glucose-6-phosphate | 6.1116 | 0.00529 | 2.2765 | 0.13838 | active - free; active - rehabilitated | 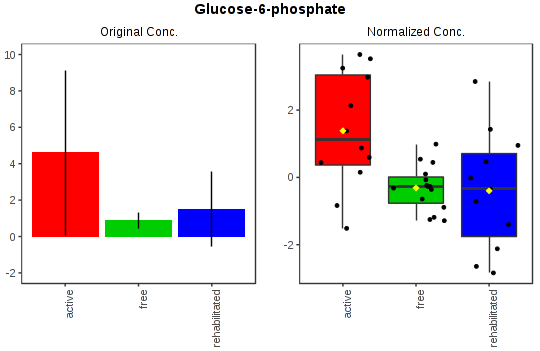 |
|  | L-Serine | 5.5104 | 0.0083082 | 2.0805 | 0.13838 | active - free; active - rehabilitated; rehabilitated - free | 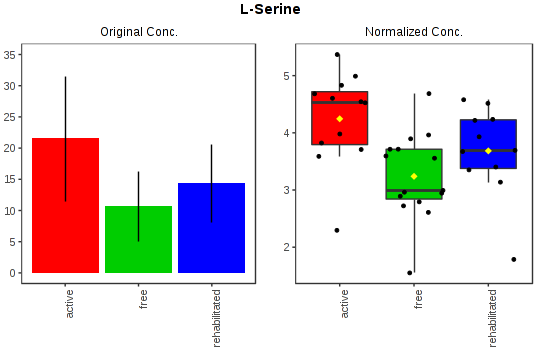 |
|  | Threonate | 5.1618 | 0.010852 | 1.9645 | 0.13838 | active - free; active - rehabilitated; rehabilitated - free | 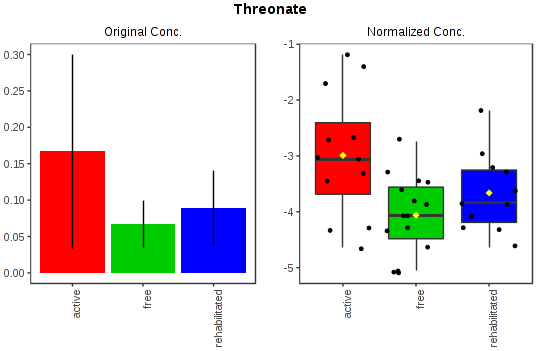 |
|  | Glucose | 5.1046 | 0.011343 | 1.9453 | 0.13838 | active - free; active - rehabilitated | 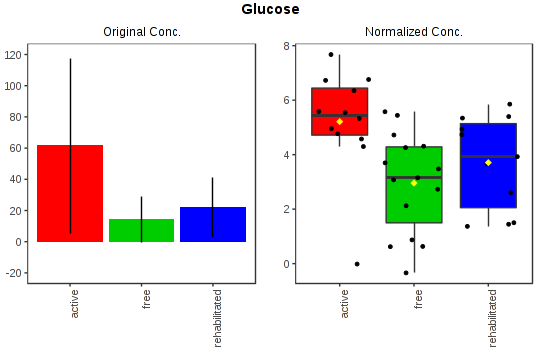 |
|  | L-Pyroglutamate | 4.1341 | 0.024447 | 1.6118 | 0.20465 | active - free; rehabilitated - free | 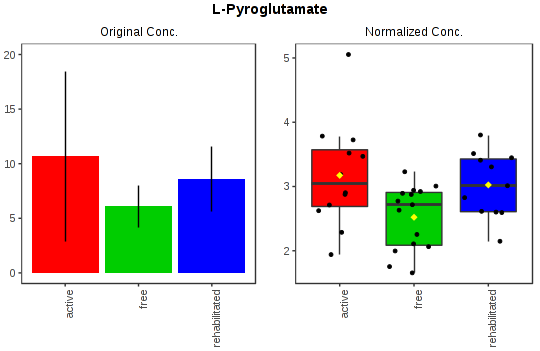 |
|  | Maltose | 4.1169 | 0.024789 | 1.6057 | 0.20465 | active - free; active - rehabilitated | 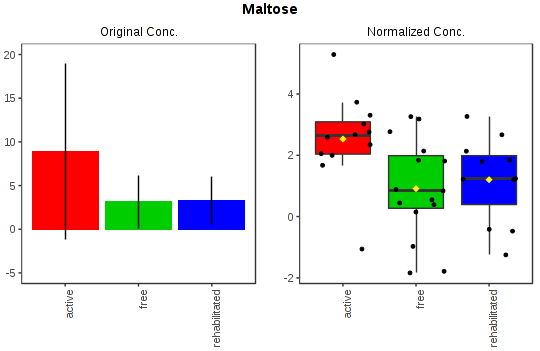 |
|  | Arachidate | 3.9788 | 0.027733 | 1.557 | 0.20465 | active - free; active - rehabilitated | 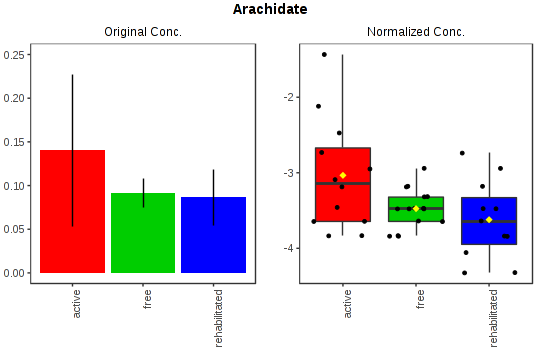 |

**Supporting table 7:** FDR adjusted analytes contributing to the small differences between the study groups “caries active” (red), “caries inactive” (green) and “caries rehabilitated” (blue) in pellicle in **figure 2**.

|  |  |  |  |  |  |  |
| --- | --- | --- | --- | --- | --- | --- |
| **Name** | **f.value** | **p.value** | **-log10(p)** | **FDR** | **Post-hoc tests** |  |
| Oleate | 4.4116 | 0.018564 | 1.7313 | 0.38985 | free - active; rehabilitated - active; free - rehabilitated | 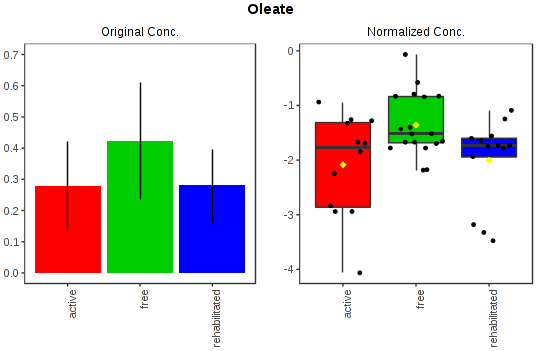 |
| Stearate | 2.3319 | 0.11017 | 0.95793 | 0.80402 | active - free; active - rehabilitated; free – rehabilitated | 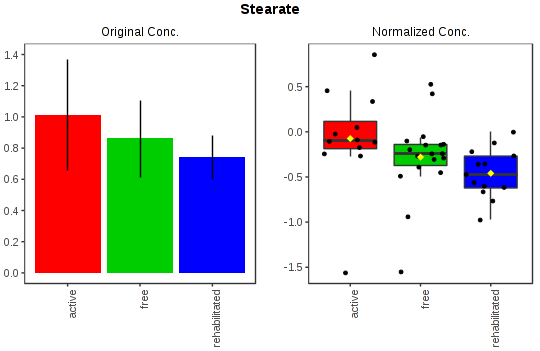 |
| Acetate | 2.2855 | 0.11486 | 0.93983 | 0.80402 | free - active; rehabilitated - active; rehabilitated - free | 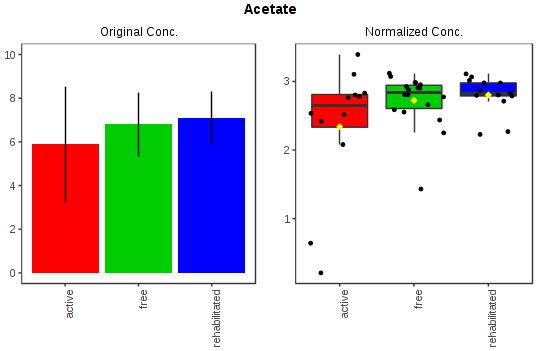 |
| Myristate | 1.8884 | 0.16456 | 0.78367 | 0.81619 | free - active; active - rehabilitated; free - rehabilitated | 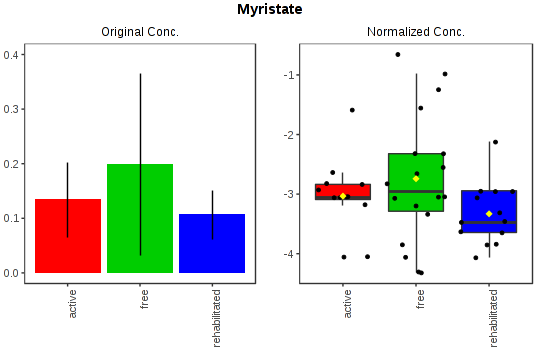 |
| Palmitate | 1.7072 | 0.19433 | 0.71146 | 0.81619 | active - free; active - rehabilitated; free - rehabilitated | 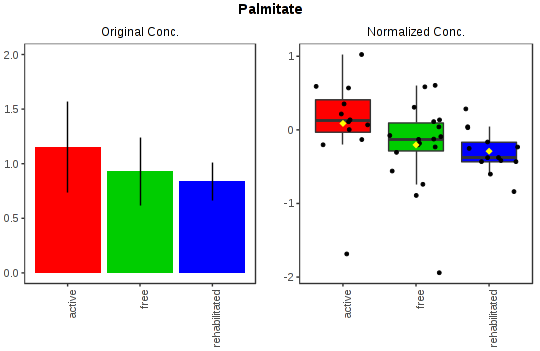 |

**Supporting table 8**: Concentrations of stable isotope labeled amino acids in the IS-solution

| Amino acid | c [nmol/mL] |
| --- | --- |
| L-Arginine*HCl (^13^C_6_, 99%) | 91.96 |
| beta-Alanine (^13^C_3_, 98%+; 15N, 96-99%) | 193.89 |
| L-Alanine (^13^C_3_, 99%) | 186.92 |
| Glycine (2,2-D_2_, 98%) | 249.74 |
| L-Asparagine*H_2_O (^15^N_2_, 98%) | 186.37 |
| L-Aspartic acid (^13^C_4_, 99%; ^15^N, 99%) | 124.65 |
| L-Glutamic acid (^13^C_5_, 99%; _15_N, 99%) | 122.48 |
| L-Glutamine (^13^C_5_, 99%) | 115.35 |
| L-Histidine*HCl*H_2_O (<5% D) (^13^C_6_, 97-99%) | 77.44 |
| L-Isoleucine (^13^C_6_, 99%) | 118.91 |
| L-Leucine (1,2-^13^C^2^, 99%) | 125.04 |
| L-Lysine*2HCl (^13^C_6_, 99%; ^15^N_2_, 99%) | 48.43 |
| L-Methionine (methyl-D_3_, 98%) | 120.54 |
| L-Phenylalanine (ring-D_5_, 98%) | 104.75 |
| L-Proline (^13^C_5_, 99%; ^15^N, 99%) | 165.00 |
| L-Serine (^13^C_3_, 99%) | 165.29 |
| L-Threonine (^13^C_4_, 97-99%; ^15^N, 97-99%) | 175.53 |
| L-Tryptophan (indole-D_5_, 98%) | 97.61 |
| L-Tyrosine (ring-D_4_, 98%) | 124.16 |
| L-Valine (^13^C_5_, 99%; ^15^N, 99%) | 127.46 |


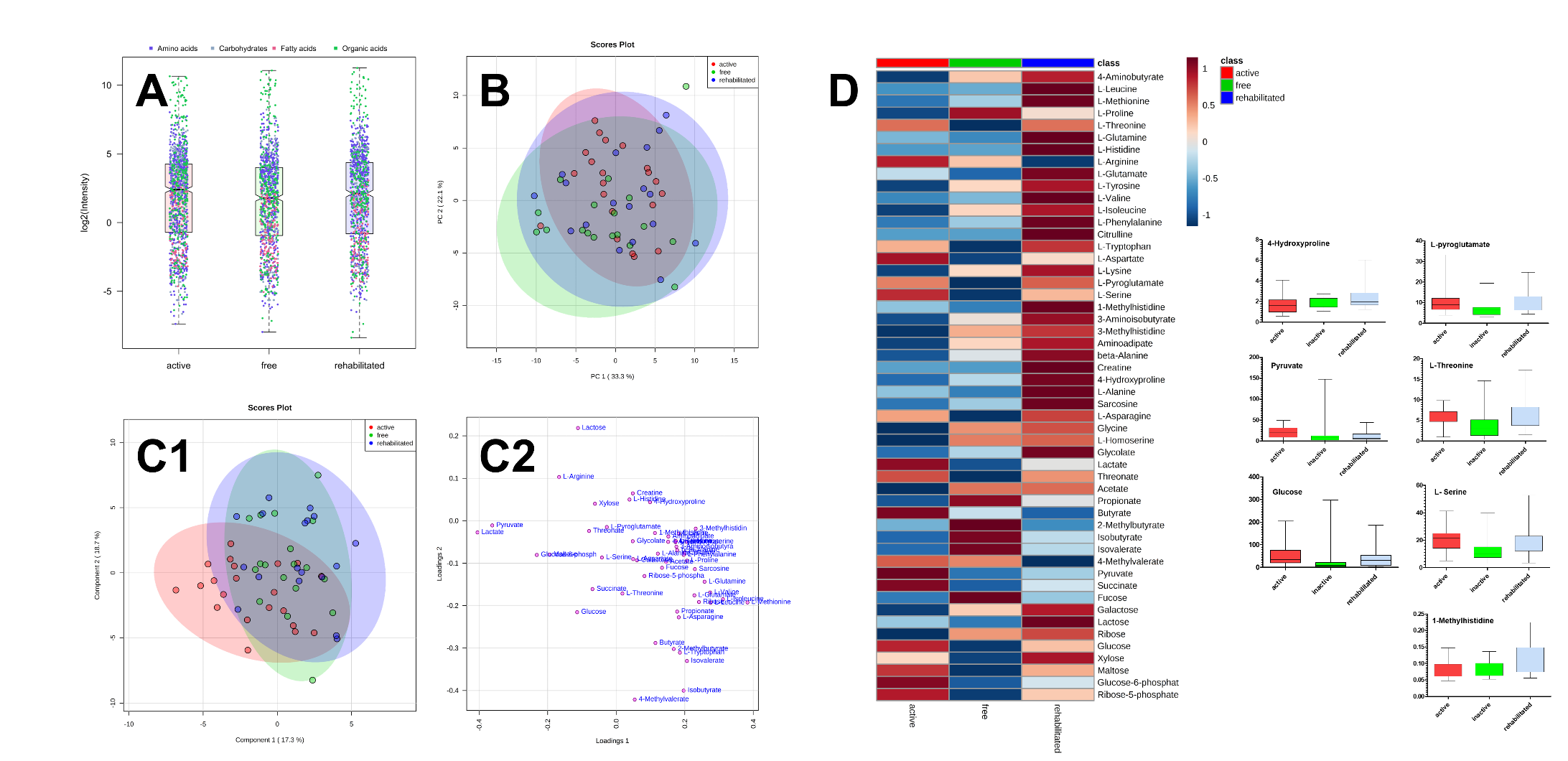


Supporting Figure 1: A) The abundance of organic acids (green), amino acids (blue), carbohydrates (grey) and fatty acids (red) in saliva is comparable in the study groups. B) A clear separation of the study groups in the PCA based on targeted data (only amino acids, organic acids, carbohydrates) is not possible. C.1) A clear separation of the study groups in the PLS-DA based on targeted data (only amino acids, organic acids, carbohydrates) is not possible. C.2) Loadings plot of PLS-DA. D) Color-coded values (raw data were “glog-ed” for normalization) of the group mean concentrations (only amino acids, organic acids, carbohydrates) and box-whisker plots (whiskers show min – max, box shows 25%, 75% and median) of the saliva concentrations (nmol/mL) of the seven top-ranked compounds (ranking based on FDR adjusted ANOVA, no significant difference between groups were detected). Data are based on n=21 caries-active, n=18 caries-free and n=18 caries-rehabilitated/medically treated children. Missing values were KNN imputed. No fatty acid data included.


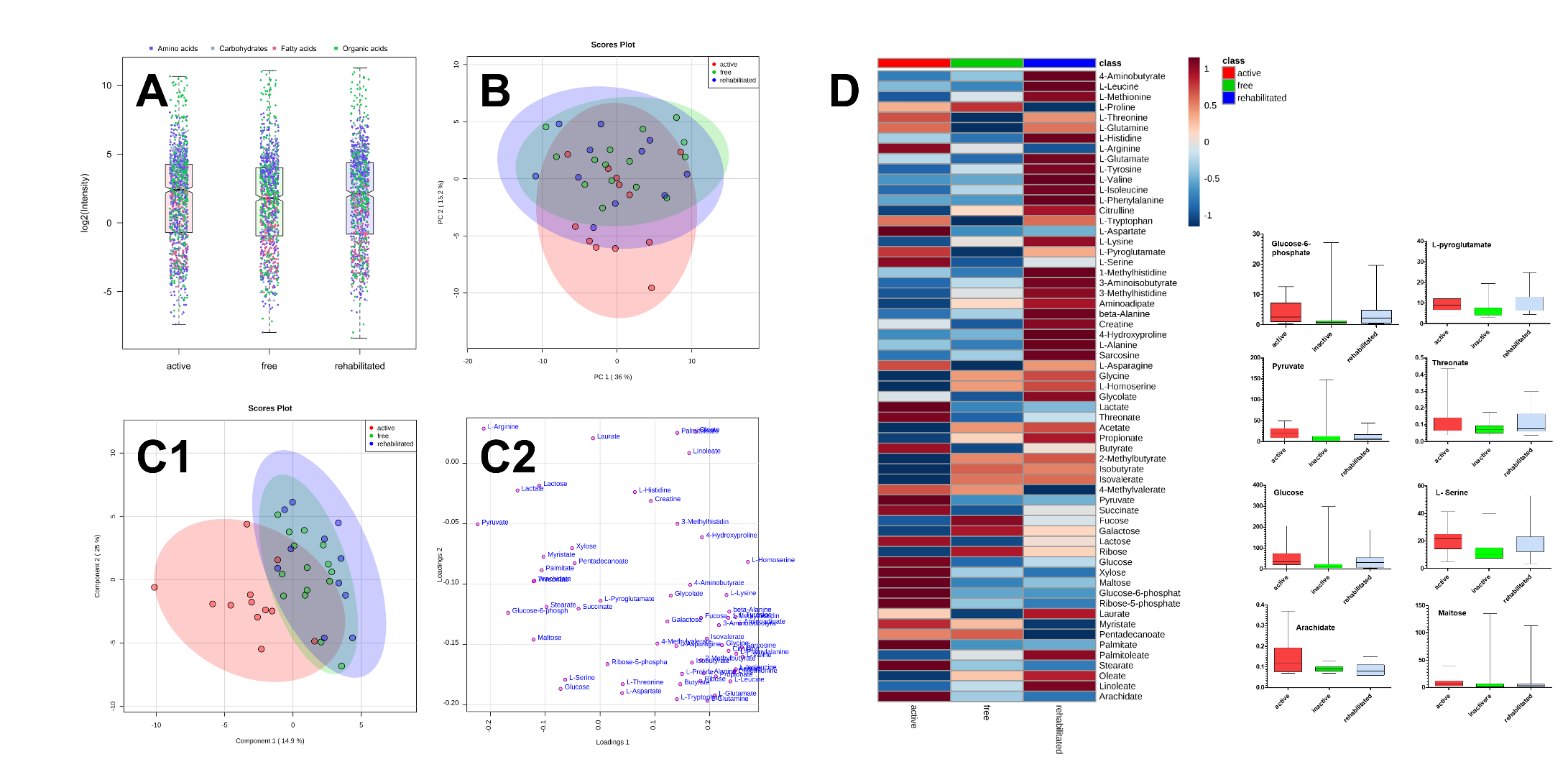


Supporting Figure 2: A) The abundance of organic acids (green), amino acids (blue), carbohydrates (grey) and fatty acids (red) in saliva is comparable in the study groups. B) A clear separation of the study groups in the PCA based on targeted data (amino acids, organic acids, carbohydrates, fatty acids) is not possible. C.1) A clear separation of the study groups in the PLS-DA based on targeted data (only amino acids, organic acids, carbohydrates, fatty acids) is not possible. C.2) Loadings plot of PLS-DA. D) Color-coded values (raw data were “glog-ed” for normalization) of the group mean concentrations (amino acids, organic acids, carbohydrates, fatty acids) and box-whisker plots (whiskers show min – max, box shows 25%, 75% and median) of the saliva concentrations (nmol/mL) of the eight top-ranked compounds (ranking based on FDR adjusted ANOVA, no significant difference between groups were detected). Data are based on complete datasets n=11 caries-active, n=15 caries-free and n=12 caries-rehabilitated/medically treated children. Missing values were KNN imputed.

**
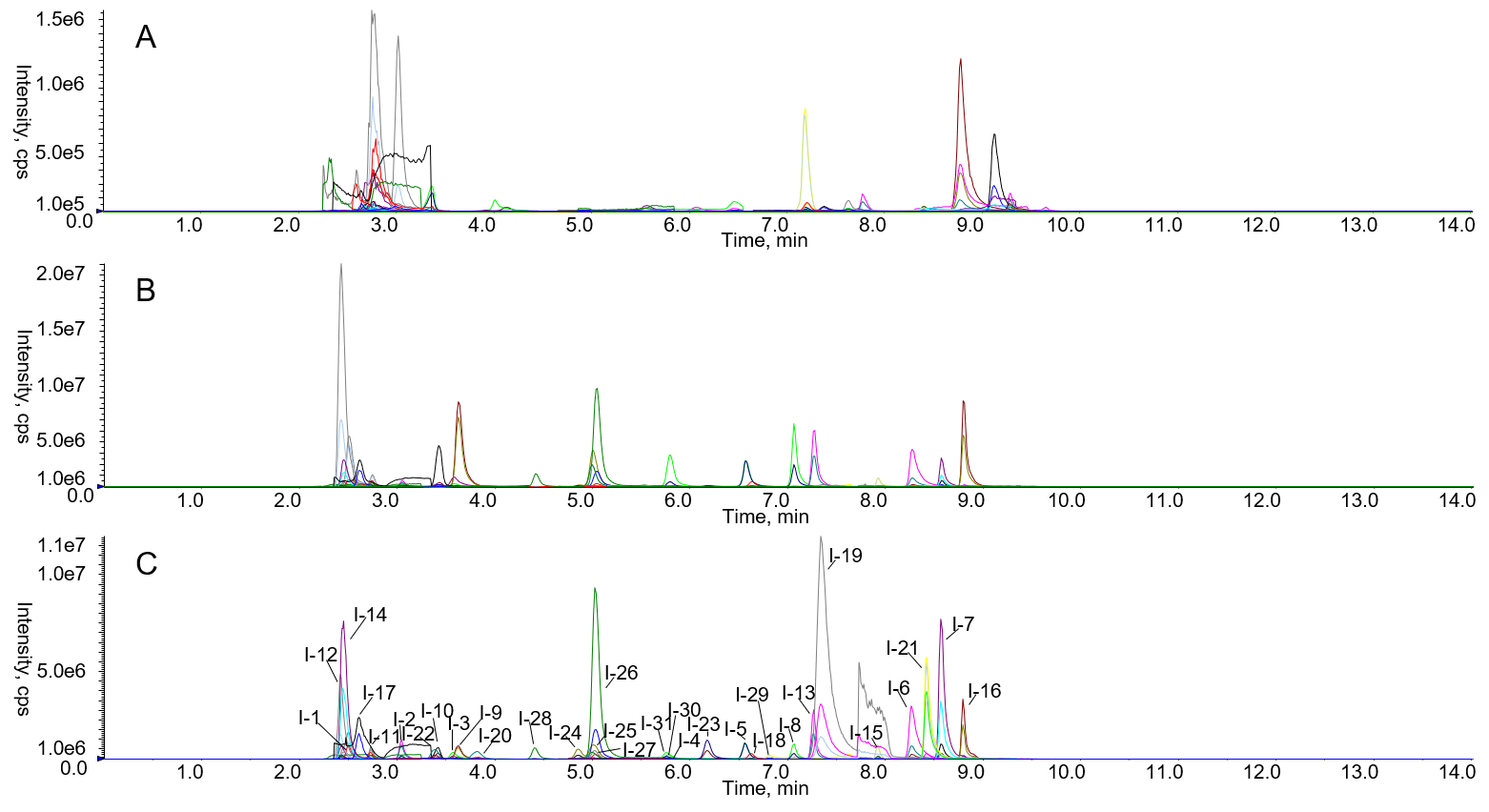
Supporting Figure 3:** Examples of LC-MS/MS(MRM)-chromatograms of amino acids and corresponding internal standards in pellicle (**A**), saliva (**B**) and standard (10 nmol/mL) (**C**). Labelling according to **Supporting Table 4**.


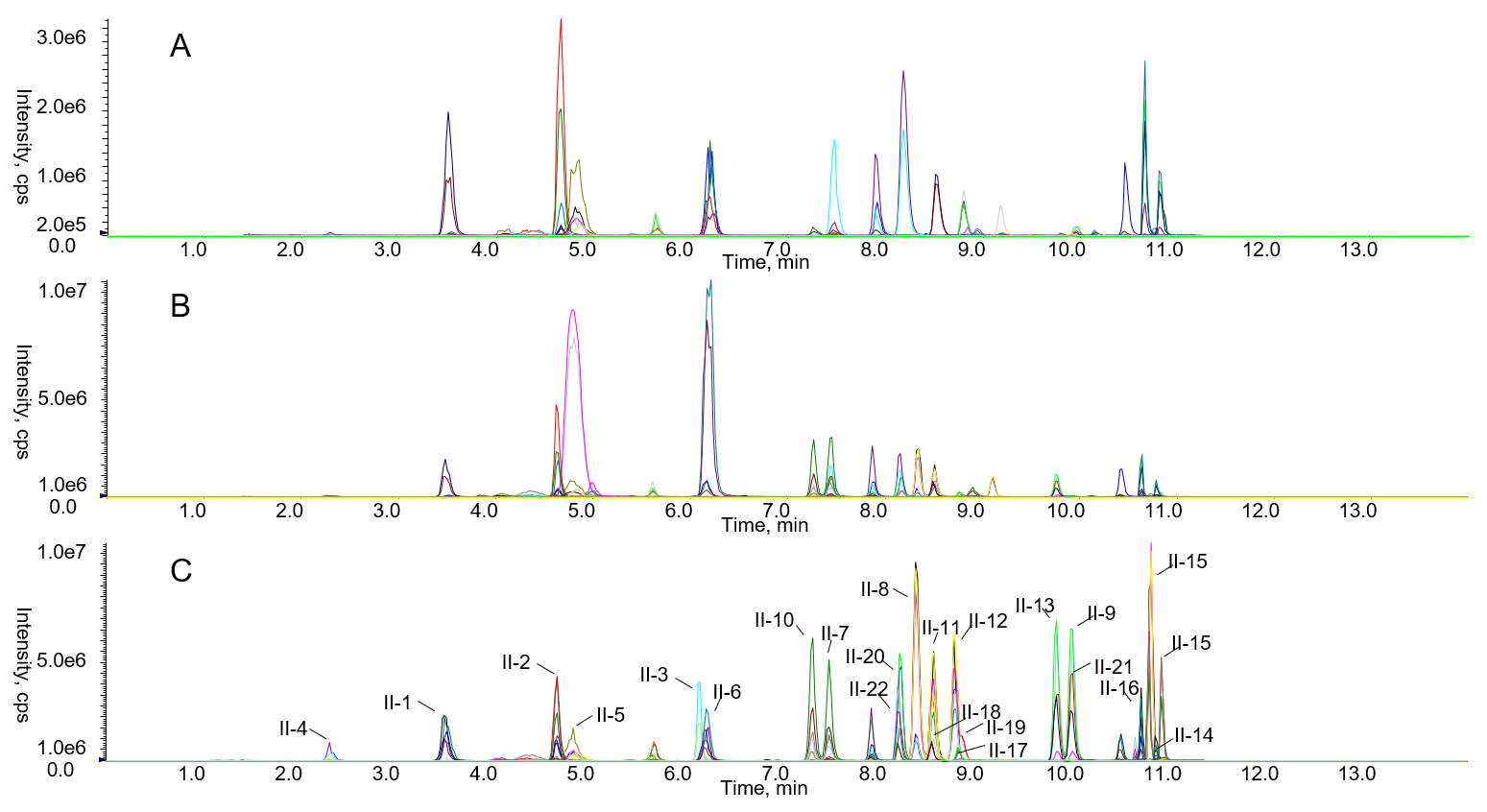
**Supporting Figure 4:** Examples of LC-MS/MS(MRM)-chromatograms of organic acids and corresponding internal standards in pellicle (**A**), saliva (**B**) and standard (100 nmol/mL) (**C**). Labelling according to **Supporting Table 4**.


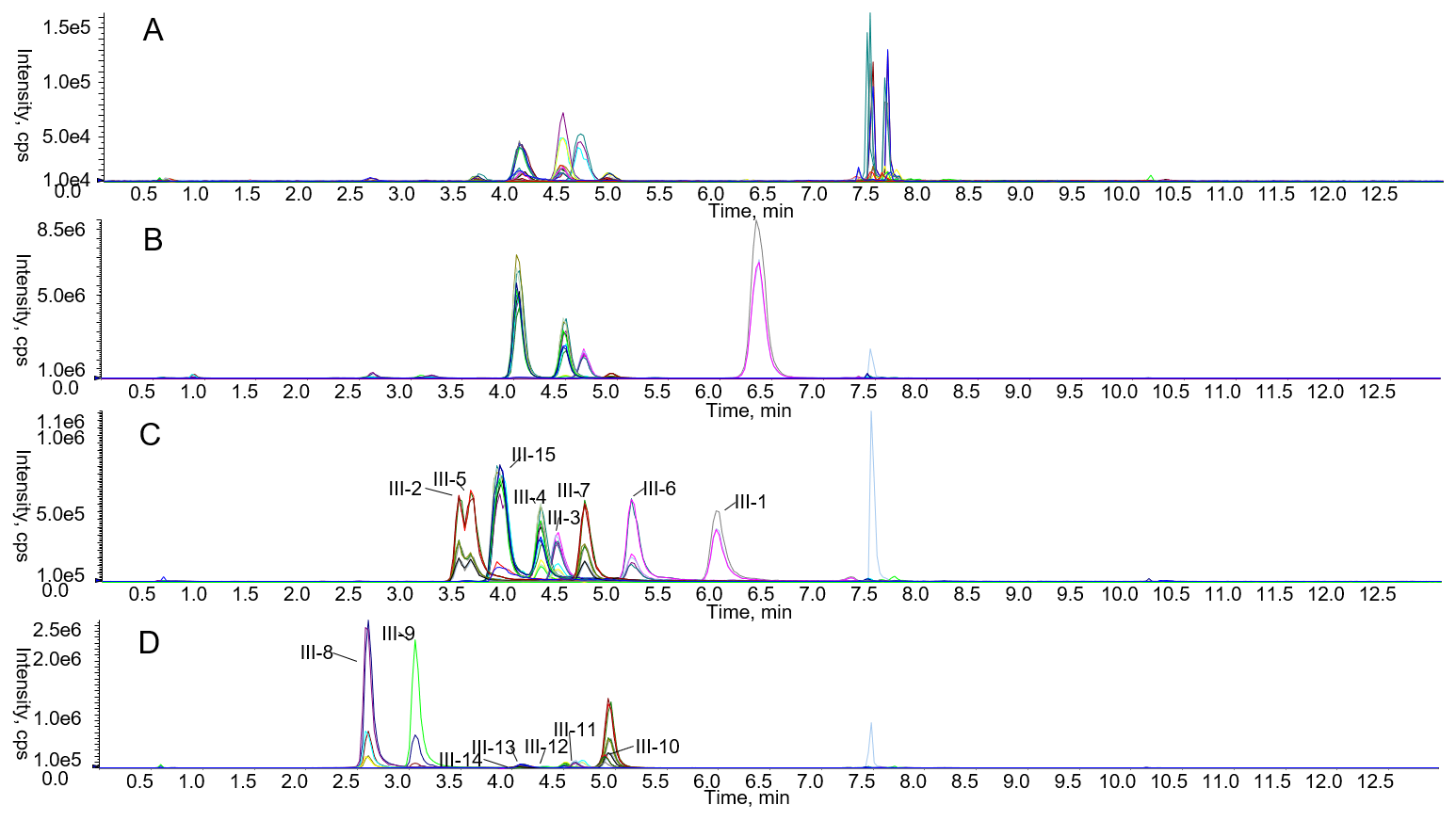
 **Supporting Figure 5:** Examples of LC-MS/MS(MRM)-chromatograms of carbohydrates and corresponding internal standards in pellicle (**A**), saliva (**B**), standard of monosaccharides and disaccharides (10 nmol/mL) (**C**) and standard of sugar-phosphates and oligosaccharides (**D**). Labelling according to **Supporting Table 4**.
